# Multi-plane attention guided 3D nnU-Net for MRI-based cervical cancer segmentation

**DOI:** 10.3389/fonc.2026.1862510

**Published:** 2026-08-12

**Authors:** Jheelam Mondal, Rajdeep Chatterjee, Mahendra Kumar Gourisaria, Manoj Sahni, Ernesto León-Castro

**Affiliations:** 1 School of Computer Engineering, Kalinga Institute of Industrial Technology, Deemed to be University, Bhubaneswar, Odisha, India; 2 Department of Mathematics, Pandit Deendayal Energy University, Gandhinagar, Gujarat, India; 3 Faculty of Economics and Administrative Sciences, Universidad Católica de la Santísima Concepción, Concepción, Chile

**Keywords:** 3D MRI cervix images, 3D-UNet model, cervical cancer segmentation, medical image analysis, nn-UNet3D model

## Abstract

**Objective:**

Over 300,000 people die from cervical cancer, which is the fourth most frequent malignancy in women worldwide. Early identification of cervical cancer has been related to much greater survival rates, and the illness is usually preventable. Accurate cervical segmentation using magnetic resonance imaging (MRI) is critical for diagnosis, treatment planning and response assessment especially in image-guided brachytherapy.

**Methods:**

In terms of 3D MRI cervical tumor image segmentation task, several cutting-edge state-of-the-art architectures are explored. We propose a novel model having MultiPlane Attention Guided Enhanced nn-UNet3D model that uses volumetric contextual information to learn spatial relationships across slices simultaneously improving border localization and noise robustness.

**Results:**

It is applied on publicly available TCGA-CESC dataset which is part of the Cancer Genome Atlas program (TCGA) and it focuses on cervical squamous cell carcinoma and endocervical adenocarcinoma (CESC). Preliminary results show the accuracy, IoU and Dice score values of 95.04%, 87.87% and 93.52% respectively.

**Conclusion:**

This study discusses the significance of MRI scans, which provide high-resolution anatomical information, improving the accuracy of segmentation algorithms in identifying and characterizing aberrant cervical tissues. Future research will concentrate on domain generality across scanners and institutions, multimodal MRI integration and clinical validation in prospective therapy scenarios.

## 1 Introduction

The fourth most common malignancy in women is cervical cancer and the treatment usually involves radiation therapy. According to the World Health Organization, around 660000 women were diagnosed with cervical cancer in 2022, while 350000 women worldwide lost their lives to the disease (1). Cervical cancer mortality occurred in low and middle-income countries. In these developing and underdeveloped nations, the low output of cervical cancer screening procedures is caused by a lack of resources and highly qualified healthcare workers. Accurate segmentation of anatomical structures and diseased regions in medical imaging is a critical step in clinical decision support, therapy planning and disease monitoring. Magnetic Resonance Imaging (MRI) in particular, provides improved soft-tissue contrast as compared to other modalities, making it an important tool in the diagnosis and treatment of malignancies such as cervical cancer. However, manual delineation of tumor volumes and adjacent organs on MRI is time-consuming, subjective and susceptible to inter-observer variability, prompting research into automated segmentation approaches (2–5). Hence, MRI is the gold standard in image-guided brachytherapy due to its superior soft-tissue contrast for delineating targets. High quality brachytherapy treatment planning requires accurate and rapid segmentation of MR data. This project’s objective is to create and evaluate deep learning models for automatically segmenting targets in high-dose-rate (HDR) brachytherapy for cervical cancer based on MR images.

Traditional segmentation techniques, such as thresholding, region expanding and deformable models, have limitations due to their reliance on handcrafted features as well as their sensitivity to noise and intensity non-uniformity. With the introduction of deep learning, particularly convolutional neural networks (CNNs), medical image segmentation has seen significant advances in performance and generalizability. CNN-based architectures like U-Net and its variants, has emerged as the standard architecture for semantic segmentation tasks across multiple modalities, including MRI, CT and ultrasound (6–8). Notably for segmentation tasks in oncology, such as cervical cancer, since they have the ability to automate and enhance diagnosis and treatment planning processes (9, 10). The cervical cancer segmentation from 3D MRI scans is a complicated but crucial step for proper radiation planning and treatment monitoring. This difficulty stems from anatomical variances, limited availability of high-quality training data, and the necessity for precise definition of tumors and organs at risk (11–13). Experiments on 3D cervical cancer segmentation using MRI images are still emerging. Mostly researchers have frequently used 3D CNNs, 3D UNet models, CNN-transformer hybrid models and advanced deep neural models combining UNet with CNN models. All these approaches highlight a clear path toward highly accurate, efficient and automated solutions for cervical cancer diagnosis and treatment planning based on MRI data. Recent research on cervical cancer have highlighted the need of automated segmentation for clinical procedures such as radiation therapy planning, which necessitates precise organs at risk (OAR) and clinical target volumes (CTV) contouring. Despite its relevance, precise segmentation remains difficult due to the varied appearance of cervical malignancies, heterogeneity in anatomical location and the typically restricted availability of annotated 3D MRI datasets. Recent initiatives have attempted to overcome these problems by applying deep learning algorithms targeted to cervical cancer MRI data, such as leveraging networks like nnU-Net trained on large cohorts of volumetric MRI images to produce effective tumor contouring (14). LANCET DIGITAL HEALTH et al. (15) has proposed an end-to-end approach that blends automated tumor segmentation and radiomics-based prognostic modeling for stratifying high-grade serous ovarian cancer patients into various risk categories. The findings demonstrate that the combined segmentation-radiomics model is able to accurately delineate tumors and stratify prognostic risk effectively, which led to enhanced prediction of patient outcomes, thus supporting its potential utility in individualized treatment planning. Miao et al. (16) in their study has presented a Swin Transformer-based segmentation framework for post-operative prostate cancer radiation. A self-supervised pretraining scheme is proposed to learn anatomical representations from limited labeled CT data and the model is fine-tuned to automatically segment clinical target volumes. The results revealed that the self-supervised Swin Transformer has outperformed the traditional CNN-based approaches in terms of segmentation accuracy and generalization, which may lead to more reliable and efficient radiotherapy planning. Dong et al. (17) has proposed cross-domain mutual learning system that is designed for fully automated diagnosis of primary tumors of nasopharyngeal carcinoma by concurrently using information from diverse imaging domains for improving tumor detection and segmentation accuracy. Its methodology is based on the transfer of mutual knowledge between domains, where complimentary characteristics learnt from one domain help the learning process of another, thus improving resilience, feature representation and generalization. The results shows that the proposed method has better diagnostic performance than the single domain or traditional deep learning methods for automatic tumor identification in nasopharyngeal cancer. Again, Wang et al. (18) in their work has used the semantic enhanced perceptual network for polyp segmentation. This process enhances the processing of colonoscopy images by fusing low-level visual information with high-level semantic features to accurately locate the polyp regions. Results demonstrate superior segmentation accuracy than standard segmentation models, with enhanced dice score, IoU, sensitivity and boundary precision, indicating its effectiveness for automated polyp detection in clinical screening.

Li et al. (19) has suggested a dual-task synergy-driven paradigm for pancreatic cancer segmentation on CT scans to increase generalization on heterogeneous datasets. This method integrates pixel classification and regression tasks by reciprocal task transformation and self-supervised learning, which can better characterize the interactions between tumor and tissue. The authors have evaluated the segmentation robustness on 594 samples from 3 datasets, and has achieved 84.07% dice for pancreatic segmentation and 9.51% improvement in cross-lesion generalization.

Several researches have focused on building and assessing novel deep learning architectures for the purpose of effective contouring and it frequently depends on the important performance measures like Dice Similarity Coefficient (DSC), Intersection over Union (IoU) and precision to assess segmentation accuracy (20–22). Hence, in our study we have also considered such metrics for evaluating the overall accuracy of the proposed model.

Following are the key points of our research contribution.

1. A multi-plane attention mechanism implementation into scratch-built 3D nnU-Net for 3D MRI segmentation.
2. The input 3D MRI volume is divided into three views: axial, sagittal and coronal, with each view processed by a separate encoder branch to learn complementing contextual characteristics.
3. Fuse all the three representations, so that the attention-guided fusion allows the network to highlight diagnostically relevant regions that may be faintly visible in a single plane, a common challenge in soft-tissue MRI.

The subsections of this study are arranged as follows: Section 2 presents the literature review, Section 3 provides materials and methods used in this study, the results and analysis are presented in Section 4 as well as the elements of the suggested study that connect to the other relevant research. The discussion section is covered in Section 5. Finally, the conclusion and future work are covered in Section 6.

## 2 Literature review

Lin et al. (23) has designed and assessed the efficacy of U-Net for the robust extraction of apparent diffusion coefficient (ADC) radiomic features and the fully automated localization and segmentation of cervical tumors in magnetic resonance (MR) images. The study involves the analysis of MR scans from 169 individuals with cervical cancer stage 1B-IVA, including diffusion weighted (DW) images. The manually contoured and fully automated segmentation techniques have a high correlation with the first order ADC radiomics characteristics. This U-Net based deep learning approach has achieved a dice coefficient of 82.00%. Wang et al. (24) in their study has developed a 3D-CNN algorithm exclusively for multimodal MRI analysis of cervical cancer. This algorithm seeks to increase the diagnostic utility of MRI images by assessing their properties using a deep learning algorithm. The study evaluated 70 patients with cervical cancer and 10 healthy persons, demonstrating the potential of 3D-CNN in this domain. The dice values achieved for whole-area tumors, enhanced tumor areas and core tumor areas are 78.00%, 71.00% and 64.00% respectively.

Gou et al. (25) in their study has proposed a multi-view feature attention network to solve obstacles in cervical tumor segmentation, such as inhomogeneous intensity, diverse tumor morphologies and restricted inter-slice correlations due to excessive slice thickness. The network sought to properly utilize 3D contextual information. The proposed MVFA-Net model has achieved DSC score value of 74.40%. SeUneter is a channel-aware U-Net designed by Zhang et al. (26) for cervical spine segmentation on MRI. It resolves the laborious and prone to mistakes nature of manual segmentation for diagnosing degenerative cervical spondylosis. The authors collected a fresh dataset of 300 patients with 600 cervical spine images information about the cervical spine, intervertebral discs, spinal cord and spinal canals in the MRI T2-weighted (T2W) modality. The proposed model SeUneter increases the network structure’s depth using the original U-Net and supplemented the double convolution feature extraction with a channel attention module. SeUneter has achieved the greatest mIoU and mDSC (82.73% and 90.66% respectively) throughout the entire cervical spine.

Kalantar et al. (27) introduced a multi-head system using dilated convolutions and shared residual connections to encode multiparametric MRI images separately in cervical cancer segmentation. The authors employed a residual U-Net model as a baseline and optimized the architecture to address image misalignment issues when merging diffusion-weighted imaging (DWI) combined with T2-weighted MRI. The dice similarity coefficient (DSC) and the Intersection over Union (IoU) for their model is reported as 81.40% and 70.20% respectively. Again, unsupervised domain adaptation (UDA) has received a lot of interest due to its capacity to perform exceptionally well on unlabeled cross-domain data. To accomplish domain adaptation, the most current UDA techniques modify image translation networks, nevertheless, the generation process might result in visual inconsistencies and inaccurate generating styles due to the instability of generative adversarial networks. To overcome this, Zheng et al. (28) presented a novel and efficient solution without image translation networks by incorporating a style improvement technique into a model based on a domain adversarial neural network (DANN). Using TCGA-CESC dataset, the dice value achieved for T1 to T2 adaptation is 56.40% whereas for T2 to T1 adaptation, dice value achieved is 65.10%. Jin et al. (29) in their work has introduced E-UNet++, an encoder-decoder deep learning architecture that incorporates an EfficientNet encoder into the U-Net++ framework. EfficientNet helps to encode multi-scale image data and hierarchical decoders with skip connections aggregate these features for accurate tumor delineation. A cohort of 228 cervical cancer patients having multimodal MRI sequences, such as T2-weighted imaging, diffusion-weighted imaging, apparent diffusion coefficient imaging, contrast enhancement T1-weighted imaging and dynamic contrast-enhanced imaging (DCE) is investigated. The findings indicate that the E-UNet++ model can reach DSC values of 68.10% - 78.60% and IoU values of 55.80% - 67.80% in different single sequences. It also provides DSC values of 64.40% - 68.70% for three DCE subsequences and all MRI sequences when combined together.

Leung et al. (30) proposed Masked-Net for cervical cancer radiation therapy, automatic segmentation of lesions and organs at risk in 3D MRI. This study focuses on the problems caused by a lack of training data and significant shape variations between and among patients for organs-at-risk. While the focus on segmentation accuracy implies particular metrics like Dice and IoU, the study has achieved a dice value of 79.00% on cervical cancer organs at risk. Rouhi et al. (31) investigated several deep learning models, including U-Net, VNet, SegResNet and SegResNetVAE for gross tumor volume segmentation in locally advanced cervical cancer using 2D and 3D segmentation. They also introduced a novel failure detection system that uses radiomic properties to improve the safety of implementation in radiotherapy operations. Xia et al. (32) developed “Cervical-YOSA”, a framework for automatic segmentation of multi-sequence MRI images in cervical cancer. In order to improve cervical tumor segmentation, this study offers two models: a fully automated model for efficiency and a semi-automatic model to refine the Segment Anything Model (SAM). The fully-automated model demonstrated strong performance with a DSC value of 85.26%, while the semi-automatic model generated a DSC value of 90.97% using a dataset of 8586 MRI slices.

Kawahara et al. (33) created an image synthesis (IS) model based on generative adversarial networks that can forecast recurrence after radiation therapy for cervical cancer that has spread locally. The Cancer Imaging Archive data is used to build T1 and T2 weighted magnetic resonance (MR) images. Radiomics characteristics from real and synthetic MR images are used to create a prediction model for recurrence after radiotherapy. The five cross-validations has achieved an average prediction accuracy of 78.90% and 74.30% for real and synthetic T1 weighted MR images. On the other hand, it has obtained 81.90% and 81.60% for both real and synthetic T2 weighted MR images. Additionally, the combined model’s average prediction accuracies for the actual T1-weighted and real T2-weighted MR pictures are 90.30%, 90.60%, and 83.80% respectively for the real T1-weighted and synthetic T1-weighted MR images. Yang et al. (34) presented “SwinUNeCCt”, a bidirectional hash-based agent transformer for cervical cancer MRI image multi-task learning. The key components of their work are- bidirectional hash-based agent multi-head self-attention method improves the interaction between local and global information in MRI, resulting in more accurate diagnosis and decreased computational complexity of the self-attention mechanism. The model has achieved an IoU value of 66.90% and DSC value of 80.20% when segmentation task did not contain any classification module. Additionally, the SwinUNetCCt model exhibits good recognition capabilities in semantic segmentation tasks using a classification module. Despite a minor increase in processing overhead and model complexity, it outperforms other comparable 3D state-of-the-art models.

Provenzano et al. (35) in their work has provided representative images that powerfully activate a model’s predictions, making complex models more interpretable particularly in image-based learning tasks. The core idea is to create simulated or saliency images to assess model explainability of recurrence prediction. This results in explainability or prediction, not guided segmentation. 27 patients who received radiation therapy are collected from TCGA-CESC dataset. A ResNet model is developed to detect recurring cervical cancer with good accuracy. The model is tested on T2W MRI data from participants with and without recurring malignancies to generate feature maps. ResNet predictions are used to determine the best predictive feature maps for each image. The top predicted features from each feature map are then combined to form a new simulated image. The resulting simulated image is put back through the initial algorithm to determine the likelihood of recurrence. This approach has achieved an accuracy of 93.00% when evaluated on T2W MRI data.

The cervical vertebral body segmentation in X-rays is the main focus of Wang et al. (36) and MRI using YOLO-U-Net proving the use of mixed neural networks for accurate segmentation in cervical regions. Images from the gathered dataset (2136 X-rays and 2184 MRIs), are annotated for this study. Four distinct image extraction models YOLOv11, DeepLabV3+, U-Net3+ for direct image segmentation and the YOLO-DeepLab network are used to evaluate the YOLO-U-Net ensemble model. This proposed model has achieved a DSC value of 91.40% and IoU value of 84.50% using MRI dataset. Cho et al. (37) has developed a new segmentation model to significantly increase the accuracy and efficiency of contouring target volumes and organs at risk (OAR) in MRI-guided HDR brachytherapy for cervical cancer. Their objective is to automate the segmentation of clinical target volumes for high-risk (HR-CTV), intermediate-risk (IR-CTV) and gross-tumor volume (GTV) critical OARs to standardize outlines and maximize treatment planning. The proposed model has achieved a mean DSC in the range of 88.00% - 91.00% and mean IoU of 79.00% - 84.00%, validating its effectiveness for accurate, automated target segmentation in cervical MRI-guided HDR brachytherapy and emphasizing its potential for real-world clinical use. Deng et al. (38) has introduced TexSem-Net, Texture Augmentation and Regional Semantic Features Fusion for histopathological segmentation of cervical squamous cell carcinoma. It uses a multiscale texture enhancement module to solve the problem of fuzzy pathological image boundaries to make discriminative morphological patterns more prominent. Furthermore, a positive definite-constrained attention method is developed to increase the multilevel fusion of texture-enhanced and semantic information for more accurate and robust cancer-region segmentation.

The following **Table 1** presents a more thorough examination of the literature survey.

**Table 1:** State-of-art articles analysis.

Author/year	Method	Dataset used	Highest accuracy	Limitations
Lin et al./2020 (23)	MRI images from 169 patients with Cervical cancer having IB-IV are used in this study. Diffusion-weighted MRI (DWI) is used in the study. The standard U-Net segmentation model is used by feeding b0, b1000 and apparent diffusion maps (ADC). Robustness of feature extraction is suggested by high correlation between automatically segmented tumors and manually segmented features	Private dataset (169 patients)	Dice value achieved is 82.00%	The testing cohort is limited to 25 patients thus undermining confidence in its generalizability to larger clinical populations. ADC maps and diffusion-weighted images are used to train the model. T2-weighted MRI images are not integrated which can provide supplementary information.
Wang et al./2021 (24)	This study aims to evaluate the value of using multimodal MRI images for cervical cancer diagnosis. Authors have developed a 3D CNN network and multimodal MRI sequences are fed as an input to it. This model results are compared to conventional CNN algorithm.	Private dataset (70 patients)	Dice values achieved are- 78% for whole area tumor, 71% for enhanced tumor area and 64% for core tumor area.	Despite multimodal MRI, the study did not optimize image fusion enough to emphasize unique features of various MRI modalities like T1, T2, DCE, IVIM etc. The model’s segmentation performance is lacking for precisely delineating intricate tumor edges. External validation on a separate dataset is not mentioned which is essential for proving practical applicability.
Gou et al./2022 (25)	This work addresses the crucial need for accurate tumor delineation in radiation planning and diagnosis by introducing a technique on MR images using multi-view feature attention network (MVFA-Net) to make most of both intra-slice and inter-slice information.	Private dataset (160 patients)	MVFA-Net outperformed eight classical and state-of-the-art segmentation networks by 2.6-11.1% in DSC.	Large MR slice thickness still reduces genuine 3D continuity in data and restricts context learning, despite MVFA-Net model’s multi-view features. Secondly, the model’s generalizability to other scanners has not been examined because it is trained on a single cohort from a single institution. Model complexity is added due to channel-wise attention fusion.
Zhang et al./2022 (26)	The suggested SeUneter model refers to as a “channel attentive U-Net”. This name implies combining a channel attention mechanism with the U-Net concept.	Private dataset (300 patients)	Mean IoU for whole cervical spine is 82.73% +- 1.59 and Mean Dice for whole cervical spine is 90.67% +- 1.31.	Just 300 patient data from a single hospital and scanner. Results obtained on single modality, single center, small dataset, 2D T2 weighted cervical MRI. Lastly, SeUneter is trained to segment typical anatomical features such as vertebrae, discs, canals and cords. Lesions or diseases such as tumors, disc herniations and stenosis regions are properly segmented in it.
Kalanter et al./2023 (27)	Authors have developed a multi-head deep learning framework that uses dilated convolutions and shared residual connections to encode T2-weighted MRI and DWI+ADC maps independently, enhancing contextual feature extraction and lowering distortions from diffusing imaging. The baseline is a residual U-Net backbone.	Private dataset (207 cases)	Best median DSC value is 82.30%.	The improvement in median DSC is not statistically significant, despite the multi-head model outperforming the baseline. External validation on a separate dataset is not specifically mentioned. The contribution of b1000 DWI channel to overall segmentation accuracy is minimal, implying that the model did not fully harness the functional contrast from diffusion-weighted imaging.
Zheng et al./2023 (28)	The authors concentrate on unsupervised domain adaptation (UDA), where labels are only accessible in the source modality and the model must adapt to unlabeled target modality data, because annotating each modality is expensive.	TCGA-CESC	T1 to T2 adaptation, dice achieved is 56.40% whereas from T2 to T1 adaptation, dice achieved is 65.10%.	Without specific architectural diagrams or ablation research on how design decisions impact performance, the style improvement and domain adaptation mechanism are explained at a higher level.
Jin et al./2023 (29)	Their work automatically segments cervical cancer on multimodal pelvic MRI. They suggested E-UNet++, a deep encoder-decoder model that uses EfficientNet-B2 as an encoder inside a U-Net++ architecture.	Private dataset (228 patients)	Single sequence DSC: 68.10%-78.60%. Single sequence IoU: 55.80%-67.80%.	All data come from one hospital which lacks generalizability. Each slice of the 3D MR volumes is split separately after being cut to 2D. It fails to take advantage of the complete 3D shape and continuity of the tumor.
Leung et al./2024 (30)	Authors have created Masked-Net, a 3D convolutional segmentation network based on a 3D U-Net with a bounding box loss, dilated layers and a masked encoder. It is improved by transfer learning from male pelvic MRI to segment cervical tumors and surrounding organs on 3D MRI for radiation therapy planning.	Private dataset (23 patients, 52 3D MRI volumes)	Average DSC of 79.00% on all anatomical structures (bladder, cervix, GTV, uterus, rectum)	Dataset is relatively small. Large inter- and intra-patient form variability in organs still presents segmentation issues.
Rouhi et al./2024 (31)	In locally advanced cervical cancer, the aim is to automatically segment the gross tumor volume (GTV) on T2-weighted pre-radiotherapy MRI and offer a failure-detection system to facilitate safe clinical use. Eight different deep learning models are evaluated for segmentation using 2D and 3D variants like U-Net, VNet, SegResNet and SegResNetVAE. A 2D SegResNet ensemble demonstrates as the best model in testing on a prospective multi-scanner population.	Private dataset (115 patients from 30 MRI scanners make up training and validation part, 51 patients from seven scanners make up the testing part.	2D SegResNet achieved DSC value of 72.00%.	There is potential for improvement especially for accurate boundary delineation in clinical radiation planning. Radiomics based failure detection may be susceptible to image variance, data heterogeneity and insufficient external validation continue to be issues. Segmentation performance is medium and study is restricted to 2D MRI.
Xia et al./2024 (32)	This study has introduced two models – Cervical-SA (semi-automatic) – here Segment Anything Model (SAM) is fine-tuned for cervical MRI segmentation and Cervical-YOSA (Fully Automated) – here a YOLO-based (You Only Look object detection backbone is integrated with SAM features.	Private dataset.	Cervical-SA model has achieved DSC value of 90.97% and Cervical-YOSA model has achieved DSC value of 85.26%.	For Cervical-SA model, insufficient prompts can deceive SAM and lower segmentation quality. It is computationally expensive.
Kawahara et al./2024 (33)	The study suggests creating MRI images (T1W and T2W) that mimic actual cervical cancer scans using a Generative Adversarial Network. By adding limited real data, these synthetic images are assessed for improving predictive models that estimate the probability of cancer recurrence following radiation therapy.	TCGA-CESC (T1 and T2 weighted)	Average prediction accuracy of real and synthetic T1-weighted MR images are 78.90% and 74.30% whereas for the real and synthesized T2-weighted MR images are 81.90% and 81.60% respectively.	One of the main drawbacks is the lack of dataset size details. Other drawbacks are general issues with GAN stability and clinical generalizability, as well as possible flaws in the quality of synthetic data for clinical prediction tasks.
Yang et al./2024 (34)	The work introduces SwinUNeCCt, a 3D transformer-based model trained on the DeepCervix dataset (dynamic contrast MRI) that has a novel bidirectional hash-based self-attention mechanism.	Private dataset (122 patients)	DSC value is 80.20% and IoU value is 66.90%.	Dataset is collected from a single hospital hence limited generalizability is present. Substantial model complexity is another drawback.
Provenzano et al./2024 (35)	This model intends to provide descriptive, simulated MRI images in order to increase the explainability of deep learning models used to predict cervical cancer recurrence following radiation therapy. A novel explainability method for simulating cervical MRI images typical of recurrent vs non-recurrent cancers is presented using ResNet feature maps.	TCGA-CESC dataset (T2-weighted MRI scans – 27 patients)	93% accuracy.	Dataset size is very small. Limited dataset used for training. Simulated visuals are indirect proxies for model decision-making, even when they provide visual sense regarding model’s emphasis. Since the method chooses the most significant feature map per image, it may oversimplify the rich multiscale representations that the deep model actually uses.
Wang et al./2025 (36)	The study focuses on the necessity of precise automatic body (C2-C7) segmentation in lateral X-ray and MRI images, a crucial step for cervical spine imaging research applications. Authors have developed a tool that automatically segments the vertebral bodies in DICOM X-ray and MRI images using YOLOv11 as backbone UNet3+ as segmentation model.	89 X-ray and 91 MRI images.	For MRI dataset, YOLO-U-Net model has achieved a Dice value of 91.40% and IoU value of 84.50%.	Dataset size is relatively small. Another drawback is longer prediction time.
Cho et al./2025 (37)	A multi-label deep model based nnU-Net is developed to segment multiple target volumes and OARs from T2 weighted MRI.	Private dataset (158 patients)	DSC value ranging between 88.00% and 91.00%.	Varied performance across structures (weak for tumor and some OARs).
Deng et al./2026 (38)	Presents a dense-prediction framework for segmentation of cervical squamous cell carcinoma by augmenting texture-level information and integrating regional semantic features.	Private dataset (Histopathology images)	–	Performance may significantly rely on the quality, staining uniformity and annotation precision of histopathology images.

## 3 Materials and methods

**Figure 1:**
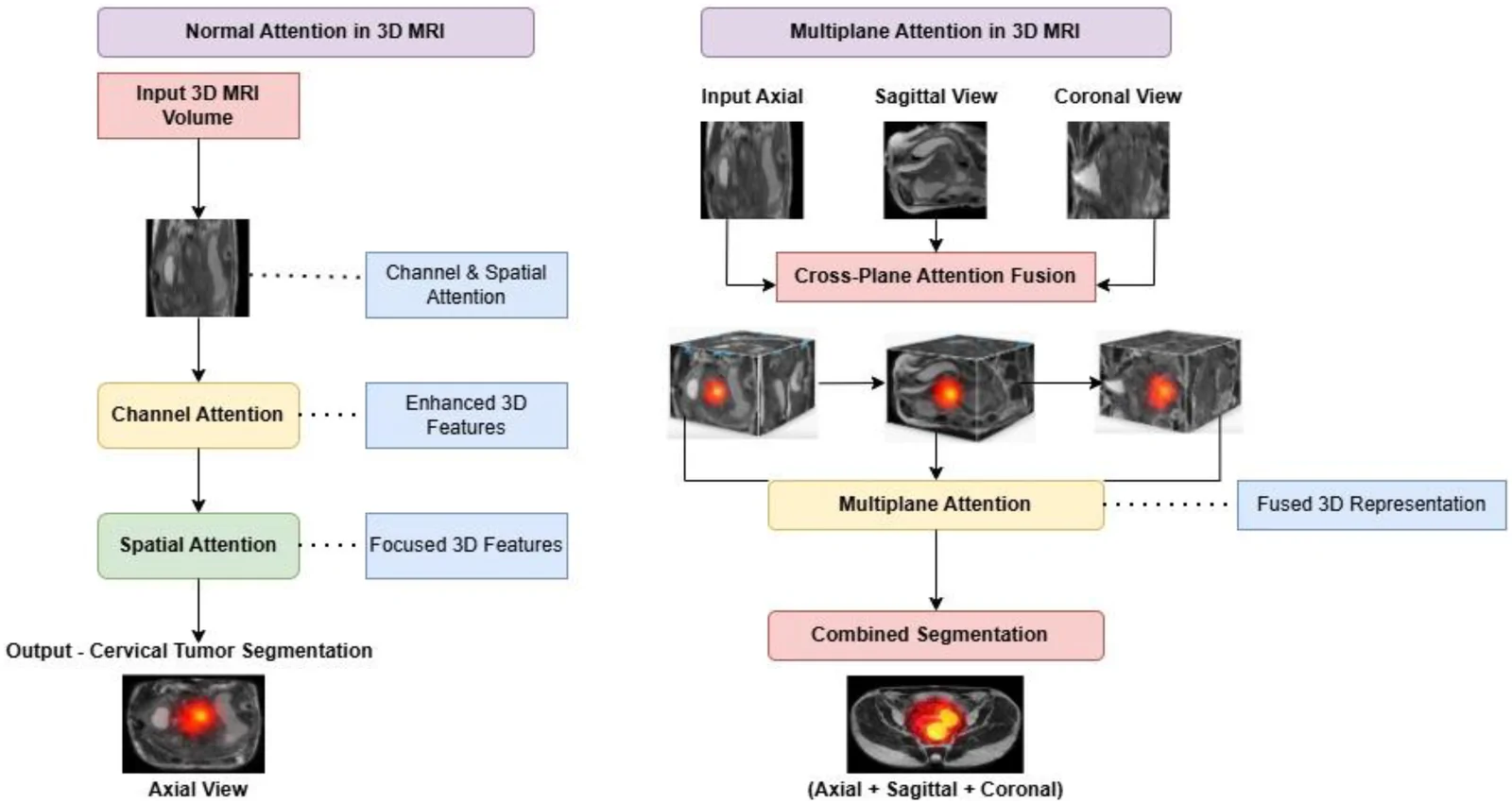
Comparison of 3D MRI attention mechanisms.

This section covers the tools and techniques used to classify images of cervical cancer. To improve computational efficiency while retaining representational capacity, our proposed neural network incorporates depthwise-separable 3D convolutions and lightweight attention methods such as squeeze-and-excitation and multi-plane context attention. The main workflow of the suggested methodology for segmenting cervical cancer using 3D MRI images is shown in **Figure 1**.

### 3.1 Normal attention versus multiplane attention

Normal Attention - The term normal attention describes CNN attention modules like –

Channel Attention: It determines which feature channels are crucial. Naturally works with 3D feature tensors and is represented in the form of B x C x D x H x W.

Where,

B represents Batch, number of 3D MRI volumes processed simultaneously in one forward pass.

C represents Feature channels, number of learned feature maps at a given layer.

D denotes the Depth or Slices, number of slices along the z-axis (superior-inferior direction in medical imaging).

H denotes the Height or Rows, number of pixels along the y-axis (anterior-posterior or vertical).

W denotes the width which is the Width/Columns, number of pixels along the x-axis (left-right or horizontal).

Equations 1–3 below represents mathematical equation for channel attention. There are three steps involved in channel attention.

Step 1 – Squeeze (Embedding Global Information) - We compress the spatial or volumetric dimensions of an input feature map X Ɛ R^CXHXWXD^ into a channel descriptor using Global Average Pooling (GAP).

(1) $${\text{z}}_{\text{c}}={\text{F}}_{\text{sq}}\left({\text{x}}_{\text{c}}\right)=\frac{1}{H\;X\;W\;X\;D}\sum_{i=1}^{H}\sum_{j=1}^{W}\sum_{k=1}^{D}x_{c}\left(i,j,k\right)$$

Step 2 - This stage uses a bottleneck structure (two completely connected layers) and a sigmoid activation function σ to capture channel-wise dependencies.

(2) $$\text{s}={\text{F}}_{\text{ex}}\;\left(\text{z},\text{W}\right)=\sigma \;\left({\text{W}}_{2}.\delta \left(W_{1}.z\right)\right)$$

Where,

*δ* is the ReLu activation and s is the vector of learned weights.

Step 3 – The original output is scaled by these weights to produce the refined feature map.

(3) $$\tilde{x_{c}}=s_{c}.x_{c}$$

Spatial Attention: Finding relevant voxels or regions in the MRI volume is known spatial attention. 3D spatial attention is extended using 3D convolutions. These are frequently employed in 3D U-Net style cervical cancer classification and segmentation. By extending attention operations to 3D, standard CNN-based attention may be easily applied to 3D T2-weighted cervical MRI. To identify the most crucial voxels, the 3D volume is compressed along the channel axis. First the data from all channels is combined in order to identify geographical dependencies. Both average pooling and max pooling is applied throughout the channel dimension for a feature map as denoted below in Equations 4, 5.

(4) $$X_{avg}=\frac{1}{C}\sum_{c=1}^{C}Xc$$

(5) $$X_{\max}=\underset{c}{\max}\left(X_{c}\right)$$

A single 3D spatial descriptor is produced by concatenating these two maps. The spatial dependencies are then learned by passing this description through a 3D convolutional layer, usually with a 7x7x7 kernel. This is depicted below in Equation 6.

(6) $$M_{s}\left(X\right)=\sigma \left(f^{7X7X7}\left(\left[{\text{X}}_{\text{avg}};{\text{X}}_{\text{max}}\right]\right)\right)$$

Where, $\left[{\text{X}}_{avg};\;{\text{X}}_{\max}\right]$ is the concatenation of the two pooled maps, $f^{7\;X\;7\;X\;7}$ is the 3D convolution operation and

σ is the sigmoid function, which maps the importance of each voxel to a value between 0 and 1.

Finally, the spatially attended feature map is created by multiplying the learned spatial attention map Ms(X) by the initial input X. Equation 7 below shows the final expression for spatial attention.

(7) $$\tilde{X}=M_{s}\left(X\right)⊗X$$

*Multiplane Attention* - In medical imaging, this typically involves employing attention across various anatomical attention across various anatomical planes; axial, sagittal, coronal. Or alternatively engaging in multi-view feature extraction across 2D slices of a 3D volume.

Both conventional CNN-based normal attention mechanisms (channel, spatial) and multiplane attention can be effectively utilized in 3D T2-weighted cervical cancer MRI. While multiplane attention combines complimentary structural information from axial, sagittal and coronal perspectives, normal attention is performed by extending attention procedures to 3D feature volumes. Below are the key differences between normal and multiplane attention mechanisms in 3D cervical cancer MRI. Each plane calculates attention as mentioned in below Equations 8–11.

Plane 1 – Channel-Spatial (C and H)

A 2D convolution and a sigmoid are used to produce a weight map after the input is permuted to highlight the height and channel dimensions.

(8) $${\text{Output}}_{1}=\sigma \left(Conv_{2D}\left(Pool\left(X_{perm1}\right)\right)\right)⊗X_{perm1}$$

Plane 2 – Channel-Depth (C and D)

To emphasize the depth and channel dimensions, the input is permuted.

(9) $${\text{Output}}_{2}=\sigma \left(Conv_{2D}\left(Pool\left(X_{perm2}\right)\right)\right)⊗X_{perm2}$$

Plane 3 – Spatial (H and W)

The height and width dimensions received the usual spatial consideration.

(10) $${\text{Output}}_{3}=\sigma \left(Conv_{2D}\left(Pool\left(X\right)\right)\right)⊗X$$

Aggregation (The Final Equation) – The final 3D-aware output is creating by averaging the findings from all three planes.

(11) $$\text{Y}=\frac{1}{3}\left({\text{Output}}_{1}+{\text{Output}}_{2}+{\text{Output}}_{3}\right)$$

The proposed Multi-Plane Attention (MPA) mechanism is inherently different from the conventional attention modules like Channel Attention, Spatial Attention, SE (Squeeze-and-Excitation), CBAM and genetic 3D attention, as it is proposed to leverage the anatomical and contextual characteristics of volumetric cervical MRI data via plane-wise feature interaction. Channel attention highlights the informative feature channels but does not explicitly preserve the directional contextual information from distinct MRI planes. Spatial attention highlights important spatial locations in the same feature map, but cannot do cross-plane contextual reasoning. While SE module accomplishes global channel recalibration, however ignoring spatial orientation and anatomical correlations between planes. Again, CBAM blends channel and spatial attention sequentially, it still functions mostly on 2D feature representations and does not explicitly learn anatomical relationships across orthogonal MRI scans. Generic 3D attention integrates volumetric information in an integral way; however it may consider all spatial directions equally, which may weaken the orientation-specific tumor characteristics and increase computing complexity.

In contrast, the suggested Multi-Plane Attention decomposes the volumetric representation into the planes that are meaningful for anatomy and learns the attention weights for the axial, sagittal and coronal views separately before fusing. This method allows the network to learn long-range context dependencies for each anatomical orientation, maintain the complementing structural cues across the planes, improve the boundary delineation of heterogeneous tumor areas and decrease information loss due by isotropic 3D aggregation. Multi-plane fusion is a good choice for cervical cancer MRI segmentation because of the anisotropic nature of cervical tumors and the variable visibility of the tumor in different anatomical planes. Axial, sagittal and coronal views give complementary structural and contextual information because of changes in resolution of MRI and tissue contrast. The model fuses all orthogonal images, therefore providing more robust and accurate lesion localization, replicating practical radiological practice.

The following **Figure 1** illustrates the normal versus multiplane attention mechanism in 3D MRI for a sample image.

Following **Table 2** shows few significant dissimilarities between normal attention and multiplane attention for 3D cervical cancer MRI.

**Table 2:** Key differences between normal attention and multiplane attention.

Aspect	Normal attention	Multiplane attention
Processing Approach	Handles a 3D volume in its entirety or as a series of 2D slices.	Explicitly handles the axial, sagittal and coronal anatomical planes independently.
Spatial Context	Captures connections from a single point of view.	Captures spatial interactions between several anatomical planes from multiple perspectives.
Computational Complexity	The computational complexity is lower as it is a single mechanism for attention.	The computational complexity is higher as it is a fusion with several attention mechanisms (one per plane).
Feature Extraction	Extracts characteristics from a single, cohesive depiction.	Captures distinct anatomical information per view by extracting plane-specific characteristics.
Inter-plane Relationships	It has restricted capacity to identify cross-plane relationships.	Here it models the connections between various anatomical planes explicitly.
Clinical Relevance	May overlook diagnostic markers unique to a plane.	Mimics the workflow of a radiologist by going over several planes.
Tumor Boundary Detection	Good for boundaries that are clear and smooth.	Better for uneven boundaries that can be seen in several planes.
Interpretability	Restricted to a single attention map.	Improved interpretability, attention may be visualized by anatomical plane.

### 3.2 Dataset description

Cervical Squamous Cell Carcinoma and Endocervical Adenocarcinoma (CESC) cohort data collection (39), the Cancer Imaging Archive (TCIA) (40) and the Cancer Genome Atlas (TCGA) (41) are the sources of the T2 weighted MR images. A total of 46 patient’s data is considered for experimental setup out of 55 patients. Since the remaining patient’s data contained only localizer data. This localizer data captures images while initial positioning of the patients and is generally of low quality. Hence such data is not suitable for deep model training and is discarded in our study. MRI is performed using three MRI imaging units named GE Medical Systems, Philips Medical Systems and Siemens. To maintain uniformity across all slices, a thickness of 5mm is considered and sagittal (side), axial (top-down), coronal (frontal) views are only taken into account for all the patient’s data. The present study uses only T2-weighted MRI scans from TCGA-CESC dataset and omits other MRI sequences, including T1-weighted and diffusion-weighted imaging (DWI). The choice of T2-weighted MRI is driven by clinical and dataset related factors. T2-weighted imaging is considered the standard sequence for evaluating cervical cancer due to its exceptional soft-tissue contrast among the tumor, cervix, uterus and adjacent pelvic structures. T2-weighted images are commonly employed for tumor imaging, local staging, target delineation and radiation planning in patients with cervical cancer. Furthermore, to maintain a uniform dataset and prevent variability due to absent or inconsistently obtained imaging sequences, the investigation concentrated solely on T2-weighted MRI volumes. Conversely, T1-weighted MRI typically offers diminished soft-tissue contrast for delineating cervical tumor boundaries and is chiefly employed for anatomical reference or evaluation of post-contrast enhancement instead of accurate tumor segmentation.

### 3.3 Dataset visualization

**Figure 2:**
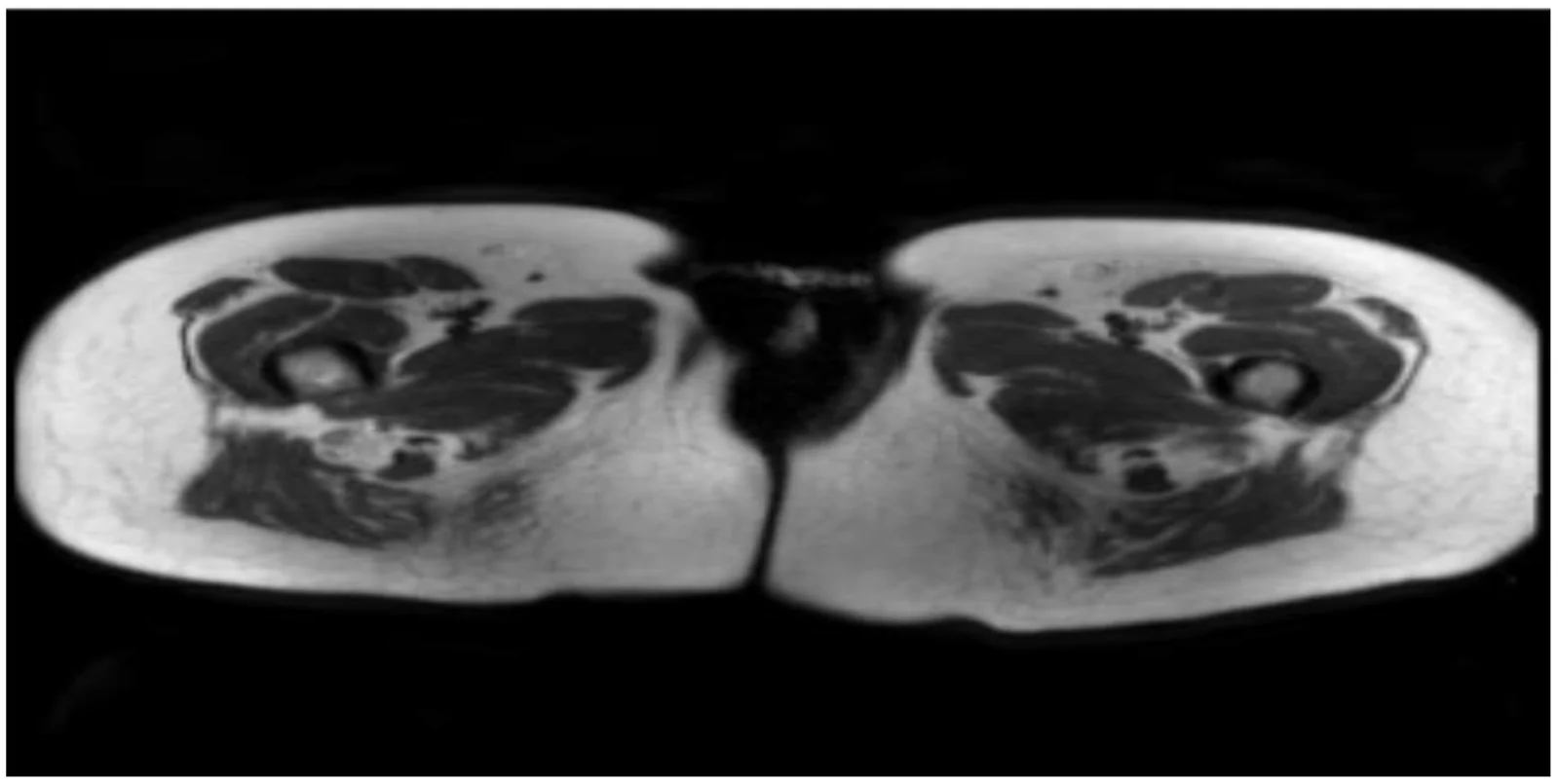
A sample DICOM image.

The original T2-weighted MRI images downloaded from Cancer Imaging Archive are in the DICOM format (Digital Imaging and Communications in Medicine). A DICOM file typically consists of two major parts: The metadata part – This contains sensitive and important information including patient information including name, ID, sex, study time, modality, slice thickness etc. And the other part is the image data which contains the real visual information. Following **Figure 2** shows a sample DICOM image and **Table 3** denotes the details of few DICOM tags of the sample DICOM image.

**Table 3:** Details of few DICOM Tags for the above sample DICOM image.

Tag Name	Value
PatientName	TCGA-VS-A8EI
PatientID	TCGA-VS-A8EI
PatientSex	F
StudyDate	20060828
StudyTime	072828
Modality	MR
Manufacturer	Philips Medical Systems
SeriesDescription	AXI T2 ssSH 5MM
ProtocolName	AXI T2 ssSH 5MM SENSE
SliceThickness	5
BitsAllocated	16
BitsStored	12

### 3.4 Splitting data

With a number of parameters, neural networks are able to memorize training data with ease, particularly when dataset is small. The model learns the patterns from the training set. The validation set evaluates how well the model applies its knowledge to data that has not been seen before. The data in our experimental setup is divided between training and validation phases in the ratio of 80 and 20 respectively.

### 3.5 Image pre-processing

Raw MRI data is provided in DICOM format and translated to NIfTI for standardized downstream processing. To accommodate different scanner exports and series formats, a hybrid technique is used, combining the dicom2nifti library with a strong custom conversion routine. This routine recursively searches each series directory for valid DICOM file and reconstructed the original anatomical ordering prior to stacking into a 3D volume. The compressed NIfTI files are stored in (.nii.gz) format and converted to 32-bit floating point format. This process ensures a successful conversion across scanners and acquisition techniques, conserved spatial calibration metadata and produced standardized NIfTI volumes suited for neural-network-based segmentation. Before proceeding with downstream processing, all converted files are visually and structurally inspected for accurate dimensions, voxel geometry and affine consistency. Hence the series-level conversion is conducted iteratively for all subdirectories within the main DICOM folder to achieve uniform, automated dataset structure.

In datasets with labels embedded in the same volume as the image, binary segmentation maps are generated using threshold-based separation (Otsu-based approach) prior to model training. When the NIfTI volume has many channels, the necessary anatomical image and segmentation channels are extracted and saved as separate 3D volumes using their corresponding affine information. Finally, the dataset is then structured as paired *_images.nii.gz and *_label.nii.gz volumes that will be loaded downstream by the neural network.

Our setup enables automatic label generation based on a threshold-based separation technique utilizing Otsu’s method. The assumption of bimodality in Otsu’s method is appropriate because of the natural bimodal distribution of intensity values in MRI volumes (e.g. tissue versus background). It reads each MRI NIfTI image and analyzes the distribution of the voxel intensities and automatically computes an optimum threshold to differentiate high intensity regions from the background or low intensity regions. The Otsu-based approach calculates the histogram of voxel intensities and selects the threshold so as to maximize the variation between two classes of intensities. Voxels with an intensity value higher than this threshold are labeled as 1, while all other voxels are labeled as 0, resulting in a binary segmentation mask. This technique removes the need of manual annotation by generating labels directly from image intensity features. The resulting label masks are then saved as individual NIfTI files, maintaining the original image matrix and header information, which guarantees spatial consistency between the MRI image and its matching label. The function also outputs label, which provides a useful measure of how much of the image volume is selected as the foreground region. Hence, the Otsu-based thresholding method offers a reproducible, automated and intensity-driven approach for preliminary label development in medical image segmentation.

### 3.6 Data augmentation

To mitigate the risk of overfitting due to restricted patient volumes, a specialized augmentation approach for 3D MRI data is implemented during training. The training volumes are subjected to random spatial flips over various anatomical axes, random alterations in brightness and contrast and introduction of Gaussian noise. These changes replicate alterations in patient positioning, scanner attributes, image contrast and acquisition noise, thereby enhancing the effective diversity of the training data. Additionally, all MRI volumes received uniform preprocessing, which includes resizing, normalization, intensity clipping and scaling to minimize inter-subject variability. The augmentations hence enhance resilience and mitigate overfitting, despite the constrained dataset size, when integrated with the patch-based learning method of the proposed Multi-Plane Attention Guided 3D nnU-Net. Below **Table 4** shows the various augmentation techniques applied.

**Table 4:** Augmentation approach used.

Augmentation technique	Values	Probability	Purpose
Random Depth Flip	torch.flip(image, [2])	50%	Improves invariance to anatomical orientation variations.
Random Height Flip	torch.flip(image, [3])	50%	Enhances robustness to spatial variations.
Random Contrast Adjustment	α = 0.8–1.2	30%	Simulated scanner-dependent intensity variations.
Random Brightness Shift	β = -0.1 to 0.1	30%	Improves robustness to acquisition differences.
Gaussian Noise	σ = 0.05	20%	Simulated MRI noise and improves generalization.

The numbers [2] and [3] represent dimension indices in the 4D tensor [C, D, H, W]. Here, C denotes channel (dim1), D denotes depth (dim2), H denotes height (dim3) and W denotes width (dim4). The width axis (left-right) flipping is omitted to maintain anatomical consistency in cervical MRI analysis. The spatial interactions among the cervix, uterus, bladder, rectum and neighboring pelvic tissues provide significant diagnostic insights. Horizontal flipping may produce anatomically unrealistic pelvic configurations and potentially affect clinically significant spatial patterns. Consequently, only physically feasible augmentations, including depth and height flips, intensity variations and noise. Furthermore, prior to augmentation, each volume is subjected to resizing to a standardized 128x128x128 volume, Z-score normalization, intensity clipping and intensity scaling to [0,1] which further reduces inter-subject and inter-scanner variability and noise addition are utilized to enhance data diversity while preserving realistic pelvic anatomy.

Below **Table 5** shows that augmentation has improved all three metrics simultaneously, not just one. This rules out the possibility of a trade-off and confirms that the augmentation technique has improved generalization rather than simply shifting bias from one metric to another.

**Table 5:** Augmentation approach used.

Metric	Without augmentation (%)	With augmentation (%)	Improvement (%)
Dice	92.75	93.52	+0.83
IoU	86.53	87.87	+1.55
Accuracy	94.58	95.04	+0.49

### 3.7 Building a scratch 3D segmentation model

We have developed a 3D encoder-decoder segmentation network inspired by nnU-Net with deep supervision. The model takes a single channel 3D MRI volume as input and produces a voxel-wise foreground probability map. The encoder is composed of five hierarchical stages. The first stage uses a normal 3D convolutional block while following stages use strided convolutions for downsampling, paired with parameter efficient depthwise-separable 3D convolutions, to reduce computational cost. Each stage includes an InstanceNorm and LeakyReLU-based double convolution block, followed by a Squeeze-and-Excitation (SE) channel attention module for recalibrating feature responses.

A Multi-Plane Attention module is used at the bottleneck and decoder to mimic anatomical context across the axial, sagittal and coronal planes. This is accomplished by aggregating channel-wise data along each plane and learning attention weights to improve informative feature channels. The decoder path mirrors the encoders with transpose convolutions for upsampling and skip-connections for spatial detail recovery. Each decoder step concatenates encoder features and applies the same efficient convolutional blocks and attention refinement. A final 1x1x1 convolution converts the result into a single-channel segmentation logit volume. Deep supervision is enabled during training by attaching auxiliary segmentation heads at various decoder depths.

**Figure 3:**
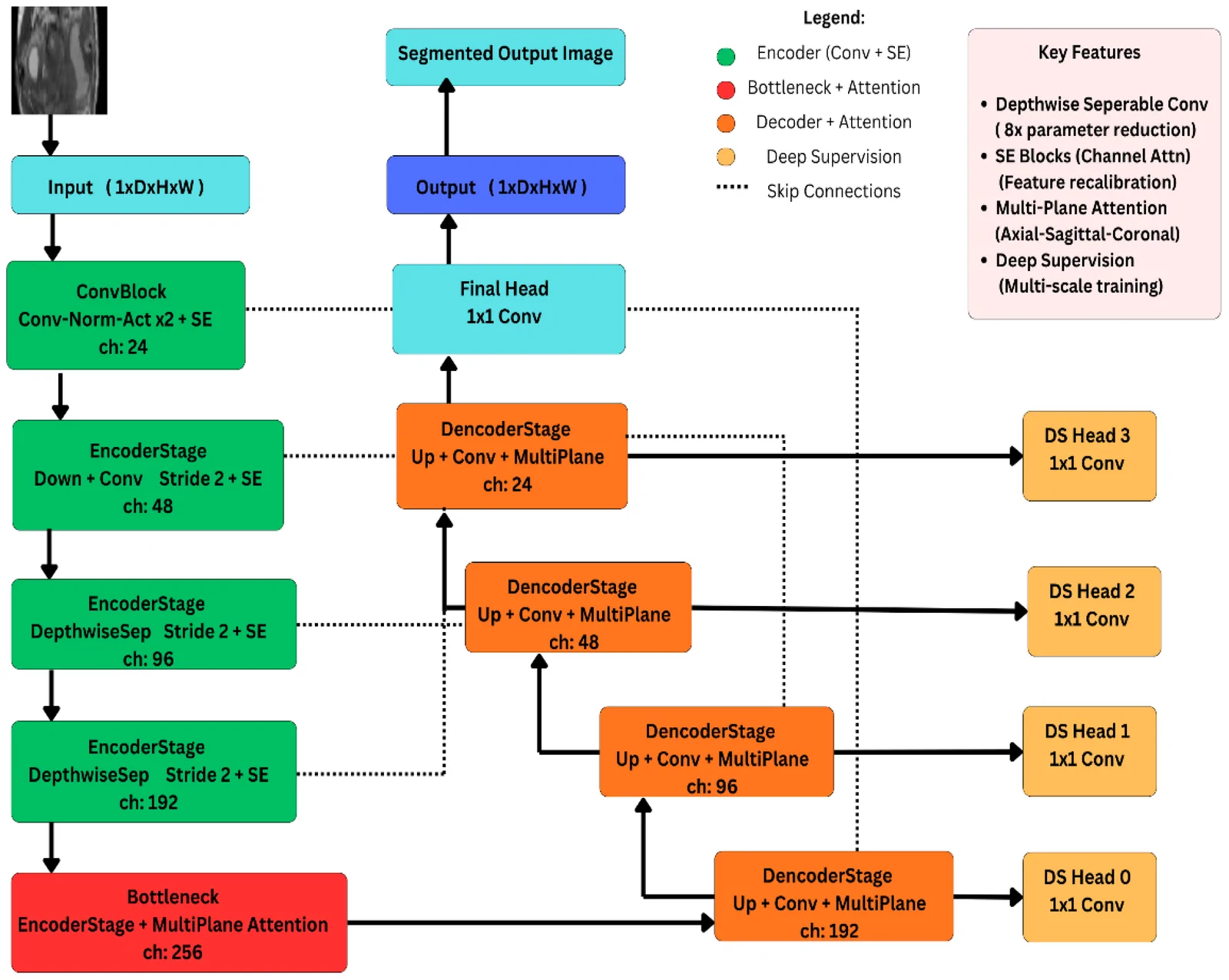
Architectural diagram of the proposed Multiplane Attention Guided nnU-Net model for cervical cancer image segmentation.

The detailed architecture of the suggested multiplane attention directed enhanced nnU-Net model is shown in **Figure 3** below. The architectural flow of this model is given below-

- Encoder Path: Progressively increasing channels in 5 stages. (24->48->96->192->256)
- Skip Connections: The path connecting the encoder features to decoder stages represented as dashed lines in above figure.
- Bottleneck: Lowest resolution with multi-plane attention. It aggregates contextual information across axial plane, sagittal plane and coronal plane.
- Decoder Path: Upsampling stages with attention modules in 4 stages. (192->96->48->24)
- Deep Supervision: For multi-scale training, segmentation heads are used at each decoder level.

The key efficiency features used in the scratch 3D segmentation model are – Depthwise Separable Convolutions which are used for 8x parameter reduction, Squeeze and Excitation Blocks that are used to apply channel attention in every Convolution block for feature recalibration, Multiplane Attention is a 3D spatial attention across axial, sagittal and coronal planes in decoder and Adaptive kernels/strides used to automatically configure based on voxel spacing.

The pseudo-code of the training network is given below in **Algorithm 1**.

Algorithm 1 **MPA-nnUNet3D with deep supervision: complete training and inference pipeline.**

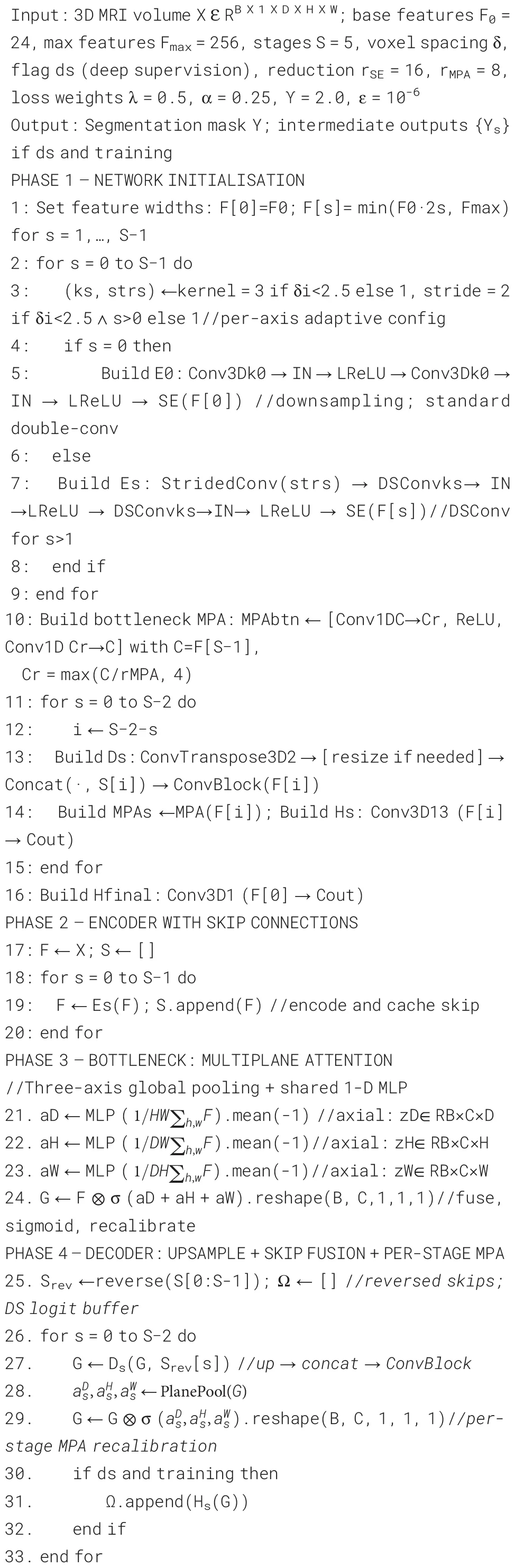

### 3.8 Software and hardware used

The experiments have been carried out in Pytorch (2.5.1) and Torchvision (0.20.1) on a Google Colab Notebook on an NVIDIA A100 GPU. The hardware included an i5 11th generation processor and 16GB RAM.

### 3.9 Model parameters

The model optimization employs a hybrid segmentation loss that includes Dice loss and Focal loss. Dice loss promotes spatial overlap between predictions and ground truth, but localized loss reduces easy negatives and enhances learning in class-imbalanced tumor locations. Deep-supervision outputs are weighted using a scale-decay algorithm. Other parameters used during training is AdamW optimizer with learning rate 1x10–^3^ and weight decay 1x10^-4^. A CosineAnnealingWarmRestarts scheduler is applied for adaptive learning rate decay. Mixed-precision training is also enabled using NVIDIA AMP to reduce memory computation. The training is carried out with a mini-batch size of 8 for 100 epochs, with an early stopping triggered after 15 epochs if no progress in the validation dice score is found. Following **Table 6** lists all the model parameters that are taken into consideration.

**Table 6:** Hyperparameters used in the proposed model.

Hyperparameters	Values
Rate of Learning	0.001
Loss Function	Dice Loss, Focal Loss
Batch Size	8
Epochs	100
Image Size	128X128X128
Optimizer	AdamW
Scheduler	CosineAnnealingWarmRestarts
Weight Decay	0.0001

## 4 Results and analysis

This section discusses the analysis and conclusions pertaining to the experimental findings.

### 4.1 Evaluation parameters used in experimental study

In 3D medical imaging, such as MRI scans for cervical cancer, segmentation entails identifying each particular voxel (3D pixel) that corresponds to the tumor. Because cervical cancers frequently have irregular shapes and poorly defined boundaries, IoU and Dice score are the most important metrics for determining how well a deep neural model’s 3D prediction matches manual ground truth contours. The most often used and recognized evaluation measures in segmentation task research are Intersection Over Union (IoU), Dice Similarity Coefficient (DSC) and accuracy (42).

Intersection of Union (IoU) – It is the ratio of the volume where the prediction and ground truth overlap to the entire volume occupied by both together. In 3D, IoU is extremely strict. If deep model predicts a tumor volume that is somewhat greater than the real cervical lesion (over-segmentation), the union (denominator part) value rises rapidly, resulting in a considerable loss in score. IoU formula is explained below in Equation 12.

(12) $$IoU=\frac{\left|A\cap B\right|}{\left|A\cup B\right|}$$

Where,

A∩B is the number of voxels where both the predicted model and the ground truth match.

A∪B is the total unique voxels identified as tumor by either deep model or the ground truth.

Dice Similarity Coefficient (Dice Score) – The dice score measures overlap but gives the intersection twice as much weight. It is the most frequently mentioned statistic in medical studies for cervical tumor segmentation. Because cervical cancers can be modest in comparison to the surrounding pelvic anatomy, the dice score is favored because it concentrates on target overlap. It is mathematically identical to the F1 score. Below Equation 13 is the formula for Dice.

(13) $$DSC=\frac{2*\left|A\cap B\right|}{\left|A\right|+\left|B\right|}$$

Where, 2∗|A∩B| is twice the overlapping volume |A|+|B| is the sum of the two volumes, ground truth volume and predicted volume.

Accuracy – In a segmentation task, the most intuitive metric is accuracy also called pixel accuracy. It simply determines the percentage of pixels in the image that are correctly classified. Below Equation 14 gives the formula for accuracy.

(14) $$Accuracy=\frac{TP+TN}{TP+TN+FP+FN}$$

Where,

TP (True Positive): Pixels correctly classified as the target

TN (True Negative): Pixels correctly identified as the background

FP (False Positive): Background pixels incorrectly labelled as target

FN (False Negative): Target pixels incorrectly labelled as background.

Loss Function – Focal loss is originally designed to reduce the weight of well-classified samples and focus on difficult cases, boosting performance on minority classes in object detection and segmentation (43). The employment of particular loss functions that counterbalance the effect of background and foreground voxels makes focal loss useful in limited datasets (44). Mu et al. (45) has conducted a thorough evaluation and large-scale comparison of around 20 segmentation loss functions on diverse medical datasets. The authors have found that neither Dice nor Focal loss alone is always ideal, while combination techniques often perform best. Such combined loss uses both overlap precision and hard-sample focus to increase segmentation accuracy. In our experimental setup, we have introduced hybrid loss during training to balance region overlap and class imbalance sensitivity. Dice loss promotes spatial overlap between predicted and reference masks, whereas focal loss reduces the weight of well-classified voxels to balance the foreground and background. Below Equation 15 depicts the combined loss function used.

(15) $$l=l_{dice}+0.5l_{\;focal}$$

Above **Table 7** displays the comparison table to justify how combined loss function is better than individual loss functions and also the final weight selection through quantitative comparison is done to strengthen the credibility of the loss function design.

**Table 7:** Loss function ablation study.

Loss function configuration	Accuracy (%)	Dice (%)	IoU (%)
Dice loss only	92.25	90.20	82.17
Focal loss only	94.79	92.92	86.78
0.1 * Dice + 3.0 * Focal (Aggressive Degradation)	95.02	93.26	87.41
0.5 * Dice + 1.5 * Focal (Mild Degradation)	94.92	93.21	87.33
Dice + 0.5*Focal (Proposed model)	**95.04**	**93.52**	**87.87**

The bold value present in last row indicates best result combination of focal loss and dice loss chosen in the experimental setup.

It is observed from the above table that our proposed model loss function configuration has surpassed all five configurations across every metric affirming it as the superior option. Dice loss optimizes the congruence between expected and actual tumor locations, addressing class imbalance at the regional level. Focal loss prioritizes difficult-to-classify and misclassified pixels, especially around tumor margins and minor lesions, while diminishing the impact of easily classified background pixels. Collectively, they enhance segmentation precision, border definition and resilience in cervical cancer MRI imaging, where tumor regions frequently occupy a significantly smaller area than adjacent healthy tissues. Our proposed model uses the combination of Dice and Focal loss which harnesses the advantages of both loss functions to tackle significant issues in medical image segmentation. The weighing factor of 0.5 preserves dice loss as the principal optimization goal while augmenting the model’s capacity to learn difficult areas. The integrated loss enhances both segmentation precision and resilience for diverse cervical cancers.

We have performed a systematic ablation across both single-loss baselines and various weight combinations (aggressive degradation and mild degradation). The weighting coefficients are empirically determined via exploratory experiments to examine the impact of overlap-based and hard-sample-focused optimization on segmentation performance. All three evaluation criteria (accuracy, dice and IoU) consistently endorse the suggested configuration robust rather than reliant on specific metrics. In the case of aggressive degradation, weight configuration used is 0.1*dice loss + 3.0*focal loss. This configuration significantly highlights challenging pixels and inaccurately classified tumor areas while predominantly diminishing the overlap-based direction provided by dice loss. Possible consequences are improved focus on hard tumor voxels, greater sensitivity to small lesions and increased risk of unstable training and has potential overfitting to boundary pixels and noisy regions. Again, in case of mild degradation, weight configuration used is 0.5*dice loss + 1.5*focal loss. Here, dice continues to provide significant region-level information. Focal loss prioritizes challenging instances. This case shows enhanced balance between overlap optimization and pixel-level discrimination. A more balanced formulation is dice loss + 0.5 focal loss. In this proposed approach, dice directly enhances tumor overlap optimization and focal loss enhances boundary and hard pixel learning. Overall, this approach gives an enhanced training stability. Experimental findings demonstrates that an overemphasis on focal loss diminishes the efficacy of region-overlap optimization, whereas a balanced combination yields enhanced segmentation performance.

### 4.2 Performance of our segmentation model

Below **Table 8** compares the segmentation performance of several 3D UNet-based architectures trained from scratch, including backbone variants (ResNet-34, EfficientNet and MobileNetv2), a baseline 3D-nnU-Net, 3D-UNet model implementation and incrementally improved versions that include Multi-Plane Attention and additional architectural refinements, and also other state-of-the-art models like EfficientNet-B2 as an encoder inside a U-Net++, YOLOv11 as backbone and UNet3+ segmentation model, Scratch VNet3D, Scratch SegResNet3D, Scratch SegResNetVAE3D. The additional performance is measured using voxel-wise accuracy, dice similarity coefficient (DSC), intersection over union (IoU), parameters and GFLOPs.

**Table 8:** Comparative analysis of our proposed model with other state-of-the-art scratch 3D models.

Performance metrics	Accuracy	Dice	IoU	Parameters	GFLOPs
UNet3D (ResNet34 encoder)	93.87	92.15	85.46	71.961M	663.415G
UNet3D (EfficientNet encoder)	92.17	90.39	82.53	11.721M	110.402G
UNet3D (MobileNetv2 encoder)	90.61	88.76	79.84	10.116M	106.072G
MultiPlane-UNet3D	75.00	70.43	54.37	2.374M	112.983G
Scratch UNet3D	93.76	91.98	85.24	13.091M	302.387G
U-Net++ (EfficientNetB2 encoder)	94.30	92.15	85.53	18.004M	515.332G
YOLOv11 as backbone and UNet3+ segmentation model	95.88	94.37	89.36	8.614M	2779G
Scratch VNet3D	95.21	93.36	87.65	43.951M	501.986G
Scratch SegResNet3D	94.62	92.43	85.98	16.510M	535.797G
Scratch SegResNetVAE3D	95.40	93.69	88.20	18.980M	174.181G
Scratch nnU-Net3D	91.47	89.51	81.01	13.496M	318.308G
Scratch nnU-Net3D + MultiPlane	91.05	89.07	80.29	13.533M	318.243G
Scratch nnU-Net3D + MultiPlane + Enhanced components (ours)	**95.04**	**93.52**	**87.87**	5.583M	291.824G

The bold values corresponding to the accuracy, IoU and Dice metrics values show the best results out of all the other cutting-edge techniques. Whereas, GFLOPs and parameter values show a moderately good result across all other techniques.

Overall, the findings strongly support the idea that combines multi-scale attention mechanisms, deep supervision and depthwise separable convolutions in the nnU-Net framework producing cutting-edge 3D segmentation performance, with the MultiPlane guided enhanced nnU-Net3D model outperforming other models present in above **Table 8** in terms of computational efficiency and representational capacity.

**Figure 4:**
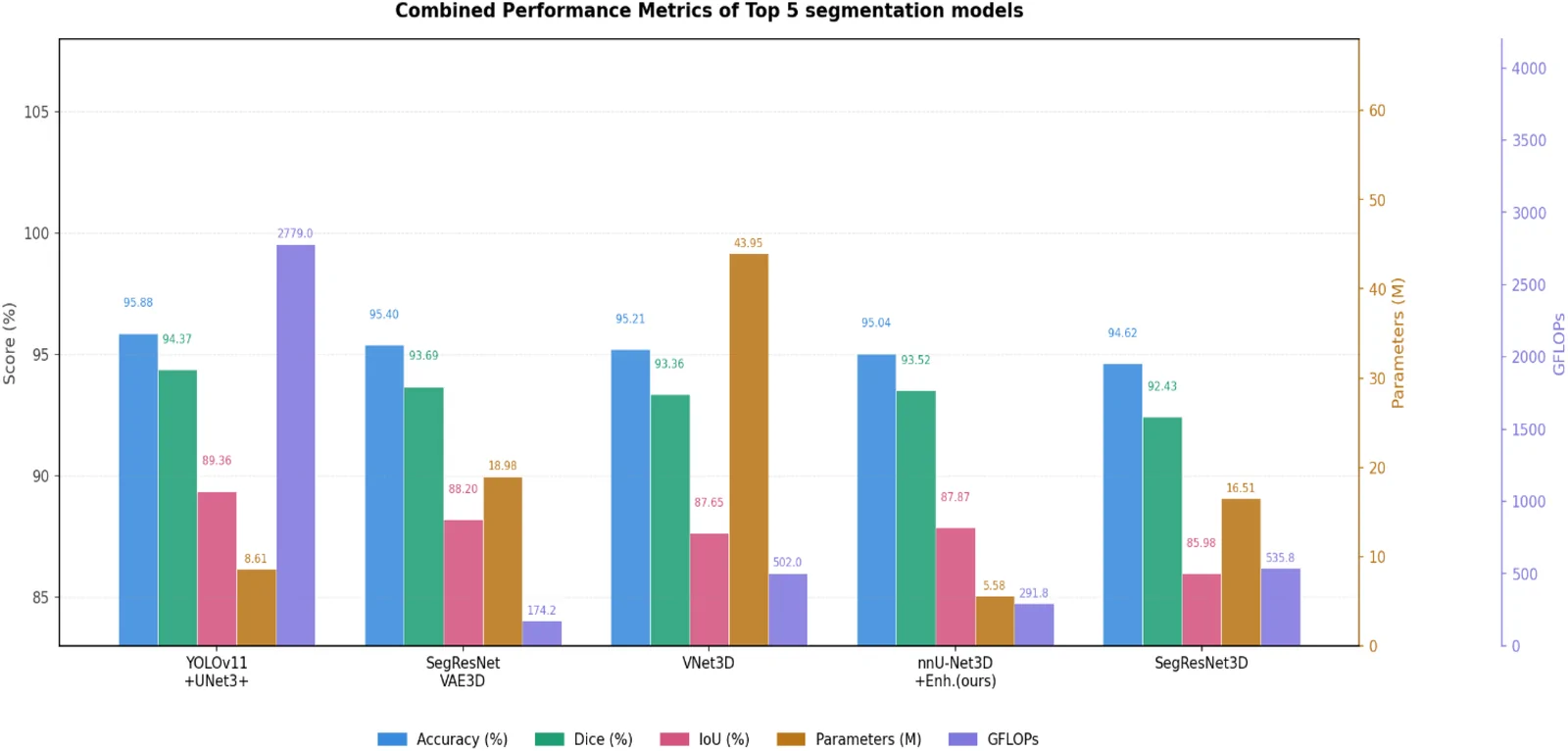
Performance of various 3D segmentation models.

**Figure 4**, generated from the above table data shows that the suggested MultiPlane guided enhanced nnU-Net3D achieves the optimal trade-off between segmentation accuracy and computing efficiency. It achieves top-2 performance on both dice and IoU, the two most clinically significant measures, while keeping the smallest parameter count of 5.583M and second lowest computational cost value of 291.824G FLOPs. The only model that beats it on raw metrics is YOLOv11+UNet3+, which uses 9.5 times more computational cost and 1.5 times more parameters, which is an impractical overhead for real-world clinical application. This demonstrates that our Multiplane integration and upgraded architectural components can effectively encode rich 3D spatial context without increasing model complexity, resulting in the most deployable, scalable and clinically viable solution among all studied architectures.

The proposed model achieves 95.04% accuracy, placing it at the top overall. It marginally edges out the closest competitor YOLOv11 backbone in UNet3+ model has achieved 95.88% accuracy. But critically, our model uses only 5.583M parameters, making it the most parameter-efficient model among all high-accuracy competitors. When comparing fairly against architecturally similar models like scratch nnU-Net3D + Multiplane attention has achieved 91.05%, the improvement is substantial, roughly 4 percentage gained by adding the enhanced components. The pretrained models like UNet3D with ResNet34 encoder has achieved 93.87% with 71.96M parameters. This is inferior accuracy value despite being 12 times larger model. Our proposed model has achieved 93.52% dice score, the highest among all 13 models. It is the most clinically meaningful metric for segmentation overlap. The YOLOv11 backbone with UNet3+ model has achieved dice value as 94.37% which is higher than our proposed model dice score, however, YOLOv11 with UNet3+ model uses a massive 2779G FLOPs versus the proposed model’s 291.82G. This means that YOLOv11 with UNet3+ model uses nearly 10x more computations, making the tradeoff strongly unfavorable for deployment. Our proposed model delivers competitive dice score at a fraction of the computational cost. Scratch SegResNetVAE3D achieves 93.69% dice value which is the nearest efficient competitor, but requires 18.98M parameters versus 5.583M parameters of the proposed model. IoU is the strictest overlap metric. The proposed model achieves 87.87%, which is second only to YOLOv11 with UNet3+ model achieving 89.36%. Crucially, this superiority of YOLOv11 with UNet3+ model comes at 9.5x higher FLOPs (2779G vs 291.82G), making it impractical for real-world 3D medical imaging pipelines. Against all other models, the proposed model clearly leads beating the next best which is SegResNetVAE3D at 88.20% while using 3.4x fewer parameters.

The reason for under-performance of MultiPlane-UNet3D is significantly due to the under-parameterized architecture having only 2.374M parameters which is the lowest value in the entire **Table 8** data. In 3D volumetric medical segmentation, characterized by intricate spatial hierarchies and precise border delineation, 2.374M parameters constitute a significant capacity limitation. This model inherently lacks the capacity to acquire significant 3D features across all planes concurrently. An IoU of 54.37% in contrast to over 80% for all other models indicates that the model is either severely under-segmenting, omitting substantial segments of the target structure or over-segmenting with inadequate boundary accuracy. This means the model acquires coarse regional features yet struggles to delineate precise borders, resulting directly from the loss of three-dimensional spatial coherence in the multiplane decomposition. This model provides exceptional computational efficiency without the need for overly large encoders while also utilizing multiplanar contextual learning, which is particularly useful for precise cervical tumor identification.

We have consequently broadened the comparative analysis to encompass notable recent methodologies specifically designed for cervical tumor and cervical structure segmentation from MRI, including Cervical YOSA model by Xia. Et al., 2024 (32) and SwinUNeCCt model by Yang et al., 2024 (34) assessed on the TCGA-CESC dataset and presented in **Table 9**. The suggested model obtains the greatest accuracy (95.04%) across all examined approaches, exceeding Cervical-YOSA (93.79%) and SwinUNeCCt (94.32%). The suggested model likewise obtains a dice score 93.52% and IoU of 87.87%, while employing just 5.583M parameters, which is much smaller than SwinUNeCCt having 25.836M parameters and merely higher than Cervical-YOSA having 4.506M parameters. These results show that the proposed framework achieves a positive compromise between segmentation performance and model compactness. We would like to say that the contribution of our work is not limited to maximizing a single overlap metric, rather, it aims to develop a lightweight and clinically practical 3D cervical MRI segmentation framework that maintains strong overall performance with reduced model complexity.

**Table 9:** Comparison with domain-specific SOTA techniques.

Author/year	Methodology	Accuracy (%)	Dice (%)	IoU (%)	Total parameters (M)	FLOPs (G)
Xia et al./2024 (32)	Cervical YOSA	93.79	91.94	85.12	4.506	2012.000
Yang et al./2024 (34)	SwinUNeCCt	94.32	92.48	86.02	25.836	359.753
Our Work (2026)	nnUNet3D+SE+Multiplane+ Depthwise Separable Conv	95.04	93.52	87.87	5.583	291.824

The ablation study is applied on baseline nnU-Net3D, addition of multiplane attention and addition of components like Squeeze and Excitation block, Depthwise Separable Convolution and is shown in **Table 10**. This study demonstrates that the architectural components in 3D MRI segmentation cannot be assessed independently; their efficacy depends on the simultaneous existence of complimentary modules. Squeeze and Excitation (SE) and MultiPlane Attention (MPA) methods are essential yet inadequate without the enhancement in representational quality afforded by Depthwise Separable Convolutions. Hence the addition of such components boost accuracy and make the model lighter, resulting in an efficient architectural design. The ultimate integrated model attains a Dice coefficient and IoU values that are indicating statistically significant enhancements compared to the baseline and incremental architectural components, thereby affirming the contribution of each component within the final suggested architecture.

**Table 10:** Ablation study of model components.

Architectural component	Accuracy	Dice	IoU
nnUNet3D	91.47	89.51	81.01
nnUNet3D+SE	90.39	83.74	75.24
nnUNet3D+SE+Multiplane	90.58	83.96	75.69
nnUNet3D+SE+Multiplane+Depthwise Separable Conv **(Ours)**	95.04	93.52	87.87

**Figure 5:**
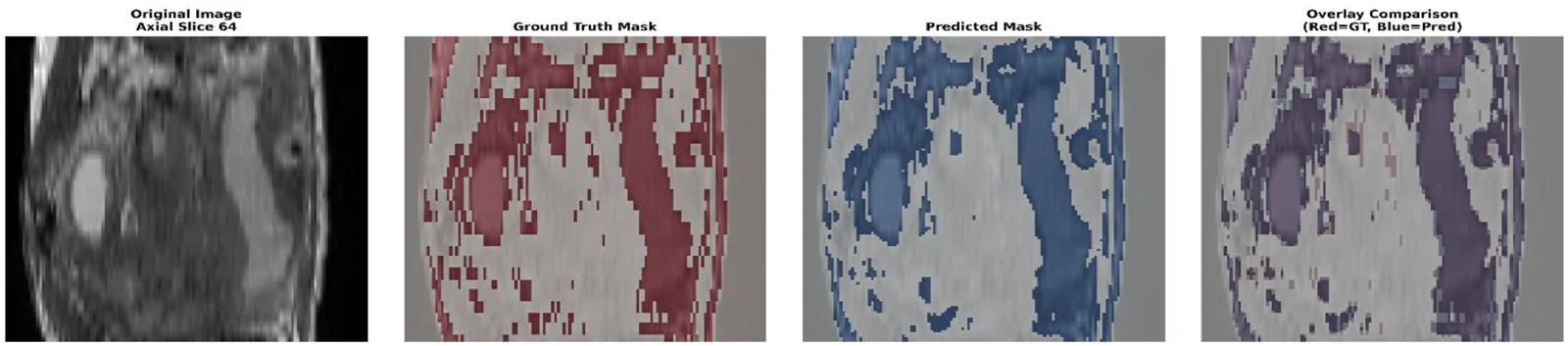
Single-slice visualization.

A qualitative comparison of the associated model-predicted segmentation and the ground-truth cervical cancer annotation on a representative axial slice (slice index 64) from a 3D T2-weighted pelvic MRI volume is shown in **Figure 5**. The initial axial T2-weighted MRI slice is shown in the first panel. The manual expert annotated ground-truth tumor mask is superimposed on the same axial slice in the second panel. Red highlights indicate the tumor area. The model-generated tumor segmentation is shown in blue in the third panel. To allow for a visual examination of agreement and disagreement, the fourth panel shows a direct overlay of both masks. Purple area is the one where the predicted and ground-truth masks overlap. The model effectively captures both the tumor core and the peripheral margins, as evidenced by the strong spatial congruence between the projected lesion and the ground-truth annotation.

**Figure 6:**
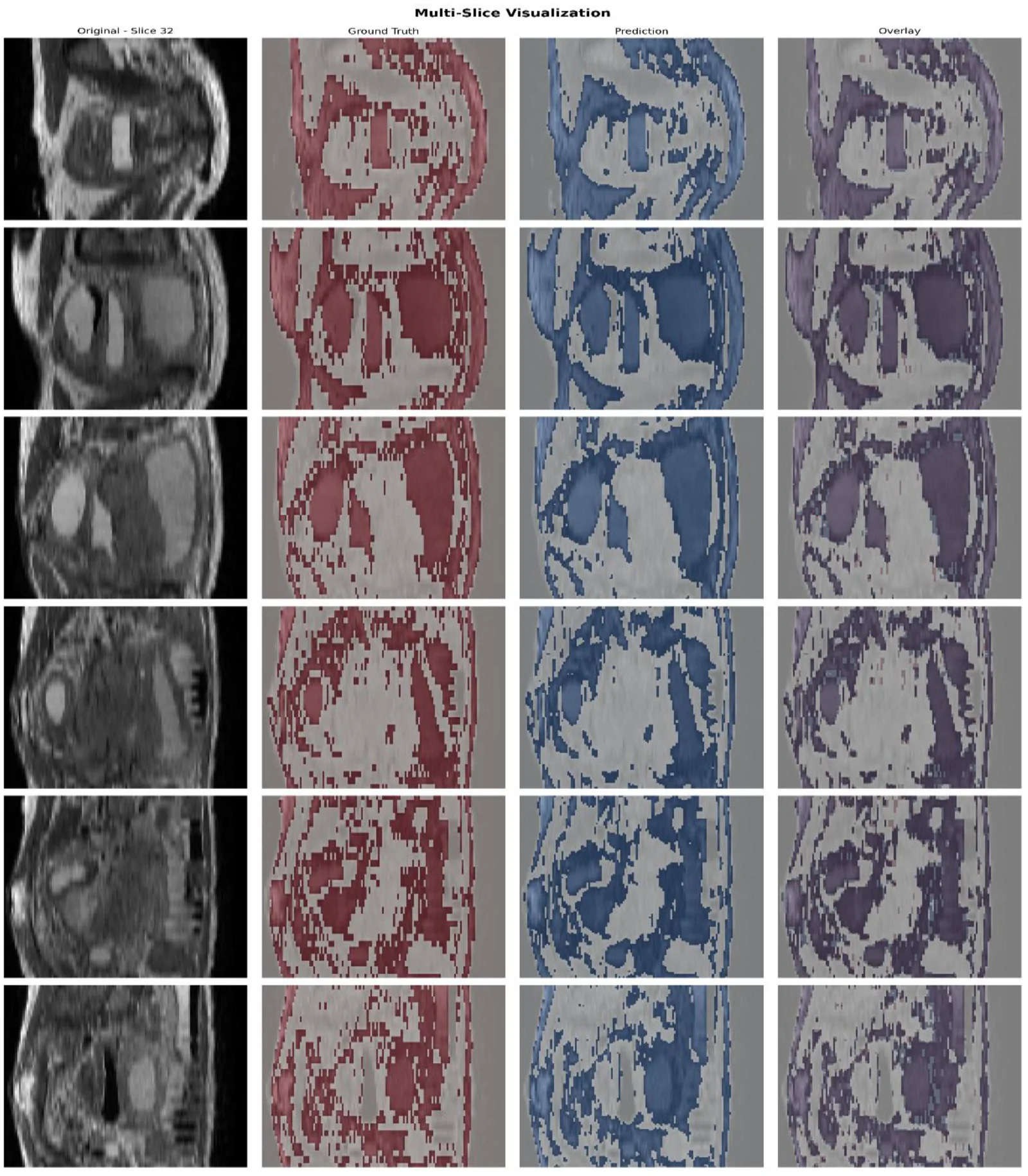
Multi-slices visualization.

Again, in **Figure 6**, the multi-slice visualization depicts the spatial behavior of the proposed model in three major anatomical planes; axial, sagittal and coronal. Representative 2D slices are collected from each subject’s 3D MRI volume and exhibited alongside the associated ground truth segmentation and model prediction. This enables a qualitative assessment of segmentation uniformity across planes and the entire tumor area. Overall, the slice-wise visualization demonstrates that multiplane attention mechanism improves anatomical awareness which is essential for radiation planning.

**Figure 7:**
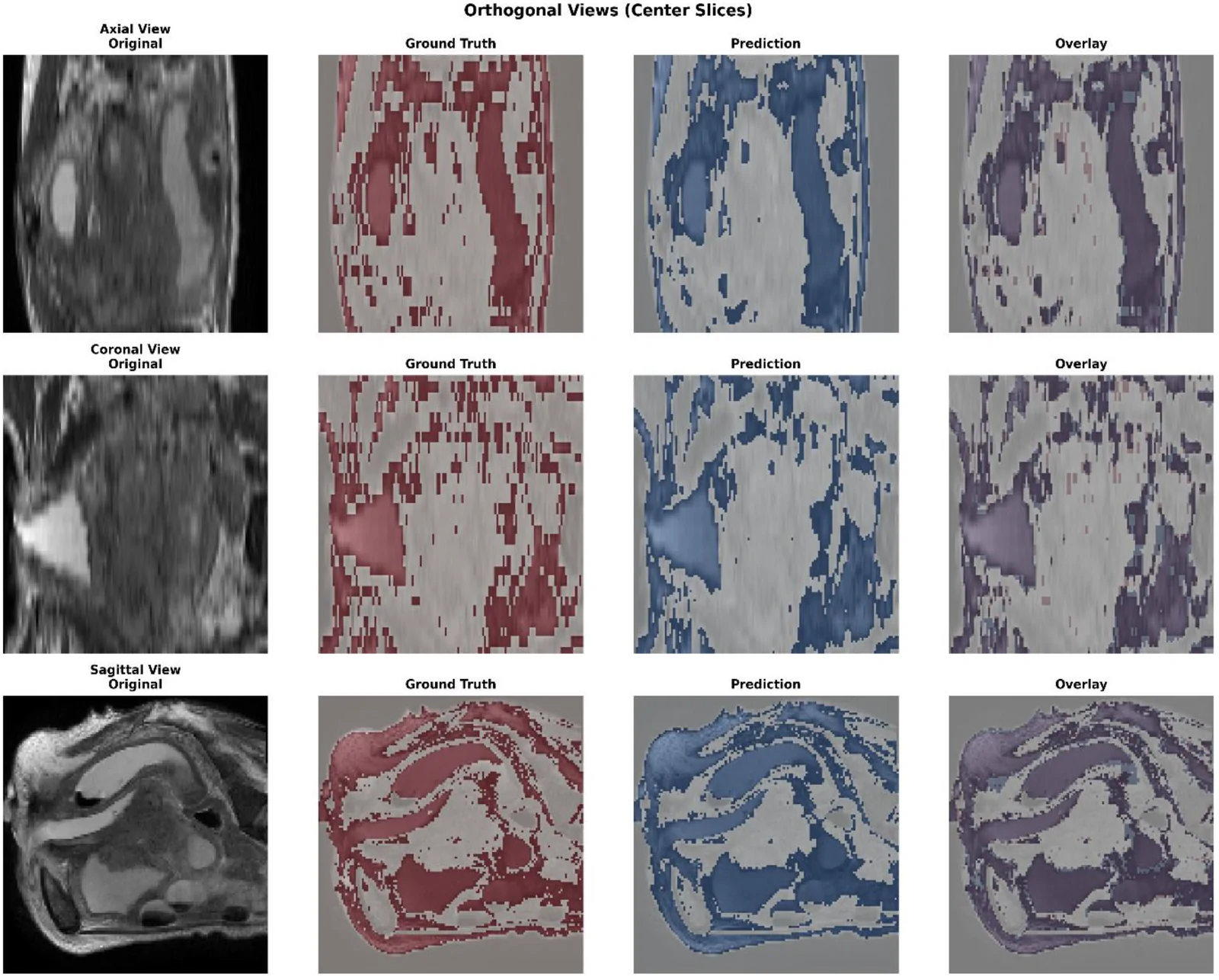
Orthogonal plane visualization of cervical tumor segmentation on T2-weighted pelvic MRI.

Using typical core slices from a 3D T2-weighted pelvic MRI volume, **Figure 7** shows the qualitative performance of the suggested segmentation model across the three primary anatomical orientations: axial, coronal and sagittal planes. The axial view, which is the most frequently utilized diagnostic orientation in pelvic MRI because it may show the cervix, parametrial tissues and pelvic sidewalls in cross-section, is represented in first row. The segmentation results in the coronal plane, which are useful for evaluating craniocaudal tumor invasion and extension patterns in relation to the uterus and pelvic floor are shown in second row. The sagittal image, which is shown in third row, offers additional details on the tumor’s anterior-posterior spread as well as its proximity to the rectum and bladder.

## 5 Discussion

### 5.1 t-statistical benchmarking of model efficiency metrics

The proposed model attains a total training time of 882s and a mean inference time of 43.99ms (std: ± 0.06 ms) for a 128x128x128 volumetric input, equating to a throughput of roughly 22 volumes per second. This indicates a decrease in inference latency. The minimal standard deviation value of 0.06ms verifies consistent and predictable GPU performance throughout multiple executions. The model meets the latency criteria for real-time clinical decision assistance and intraoperative guiding systems at less than 44ms per volume, while its high throughput facilitates efficient large-scale batch processing in hospital screening procedures. Again, the model comprises merely 5.58 million trainable parameters, signifying a huge decrease in model complexity relative to current 3D volumetric segmentation frameworks. Despite the significantly reduced parameter count, the model retains competitive segmentation performance, indicating that the suggested architecture strikes an appropriate compromise between computational efficiency and representational capacity. The suggested model utilizes merely 1.54GB of peak GPU RAM for a 128x128x128 input volume which stands at the first position out of all SOTA 3D segmentation architectures. This renders the model deployable on commonly accessible mid-range GPUs, including clinical workstation-grade equipment, without necessitating high-end infrastructure. This little memory usage immediately facilitates practical clinical implementation. Also, to assess the appropriateness of the suggested model for practical implementation, we have measured the inference latency per patient volume on an NVIDIA GPU. The proposed Multi-Plane Attention Guided 3D nnU-Net requires around 4.3s to process an entire T2-weighted 3D cervical MRI volume (128x128x128 voxels). The diminished inference time is due to the implementation of depthwise separable convolutions, efficient attention methods and a refined encoder-decoder architecture. The results demonstrate that the proposed model deliver precise tumor segmentation with computational efficiency appropriate for clinical decision-support applications.

**Figure 8:**
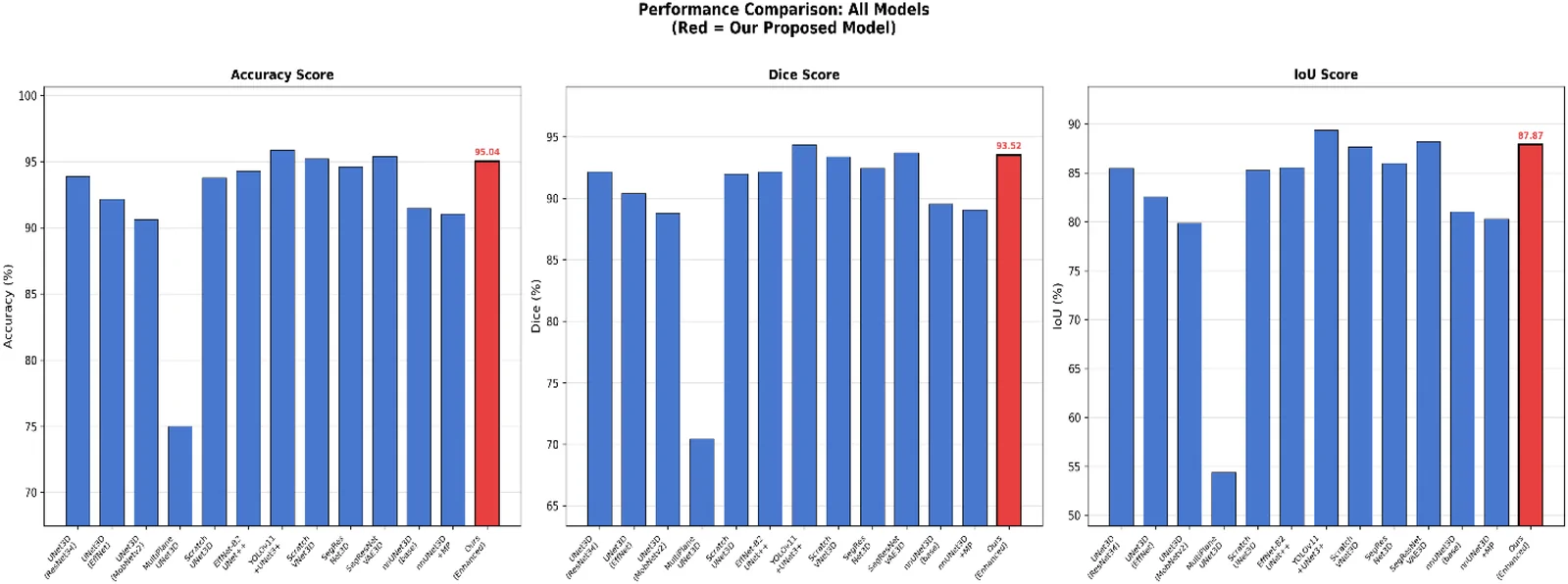
Performance metrics comparison across all models using paired t-test technique.

**Figure 9:**
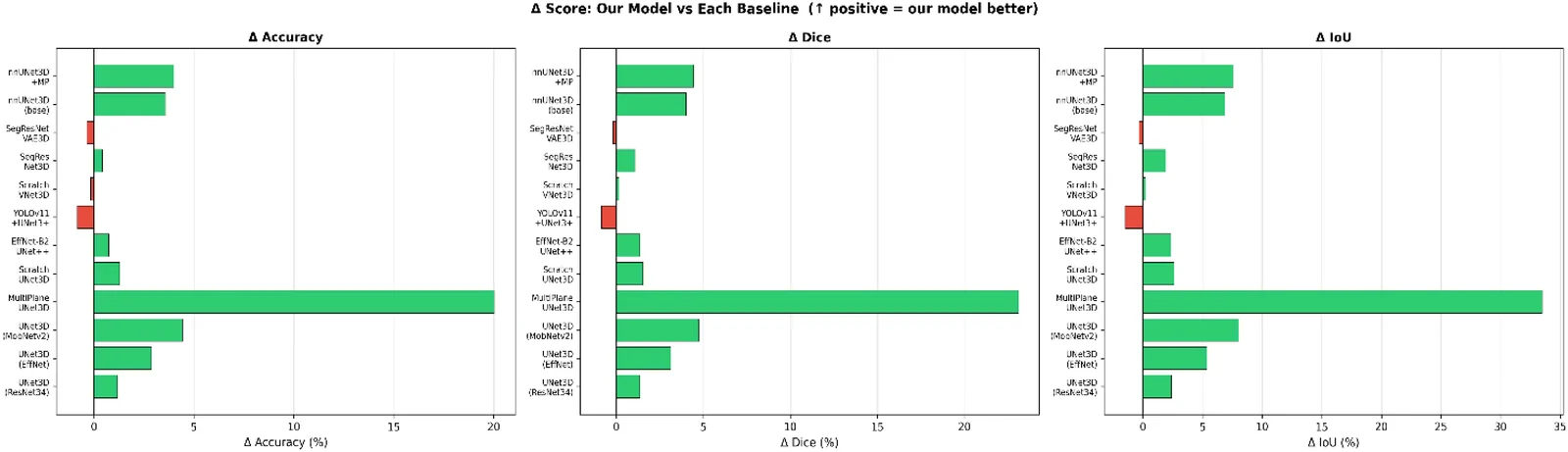
Impact on performance metrics using our model versus other SOTA models.

**Figure 10:**
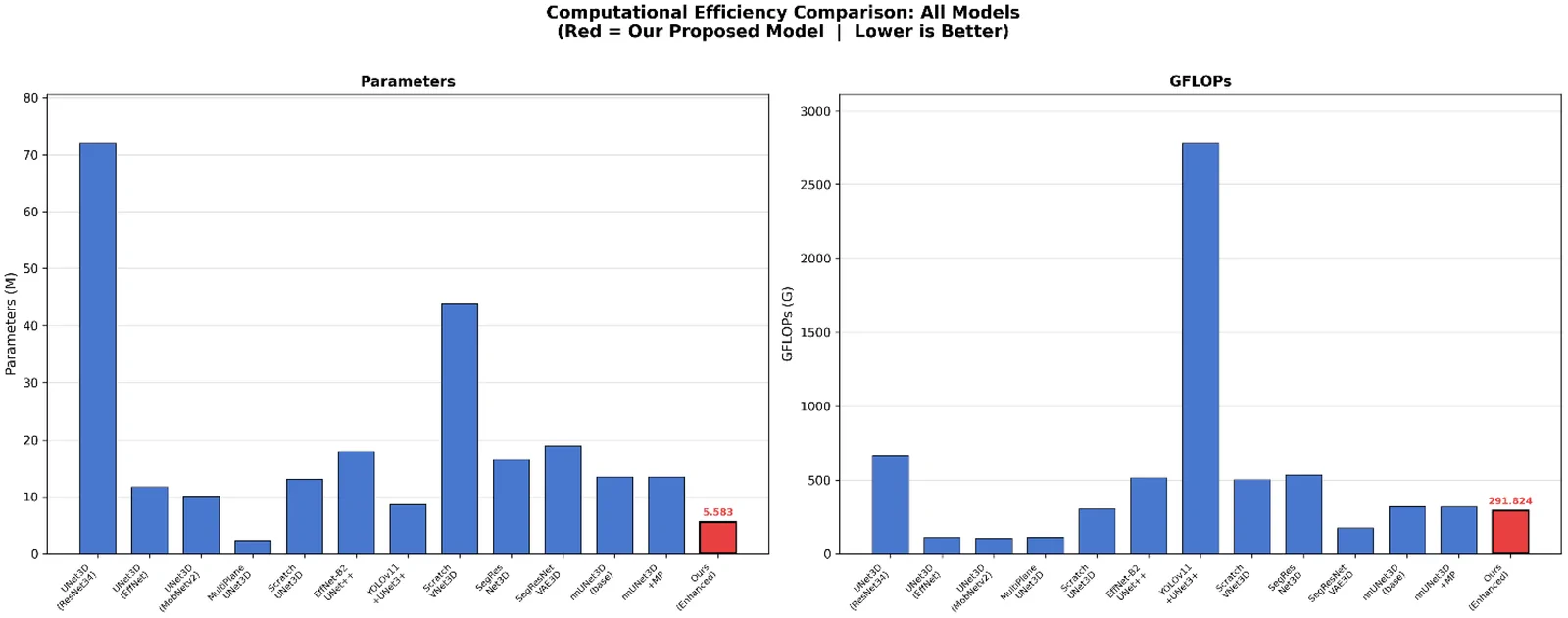
Computational efficiency comparison using paired t-test technique.

**Figure 11:**
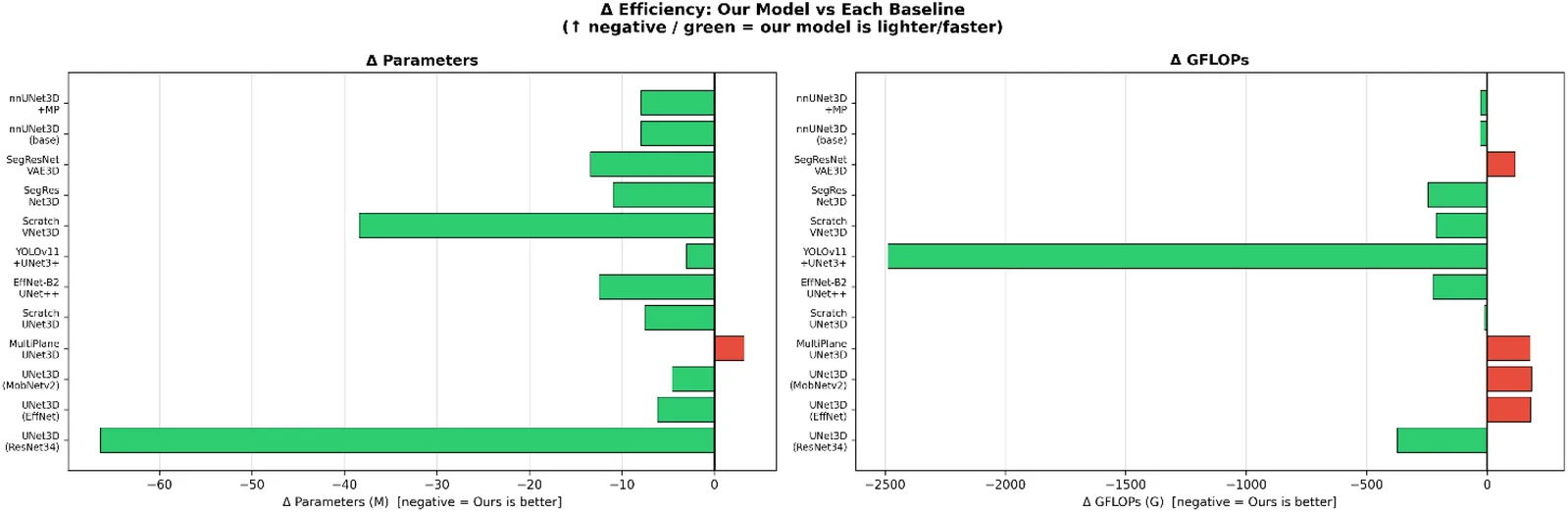
Efficiency score of our model versus other SOTA models.

Cohen’s standards stipulate that impact sizes over 0.80 are classified as significant, signifying considerable practical enhancement. The analysis demonstrates statistically significant superiority (p<0.05) over all lower performing baseline models regarding dice and IoU measures, with the associated Cohen’s d values largely falling within the large effect range. This indicates that the enhancements are both statistically significant and clinically relevant. Following **Figures 8**–**11** show the paired t-test results across the performance metrics (accuracy, dice and IoU) and also on computational efficiency parameters (GFLOPs and total model parameters).

**Figure 12:**
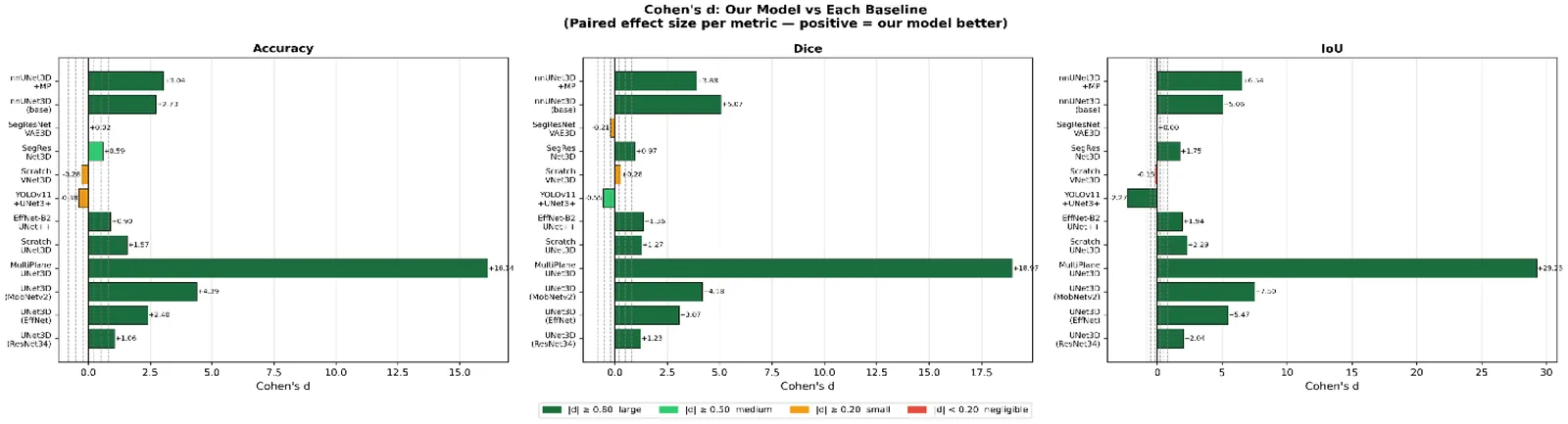
Cohen’s d test result of our model versus SOTA models across accuracy, IoU and dice.

Cohen’s d values indicate statistically significant big effect sizes (|d| > 0.80) for our model compared to most baselines across all three metrics. **Figure 12** demonstrate the sole negative d values are associated with top-performing models (YOLOv11+UNet3+, SegResNetVAE3D, VNet3D), where the differences are minimal (|d|< 0.55), suggesting that our model remains highly competitive even when it does not achieve the highest rank.

**Figure 13:**
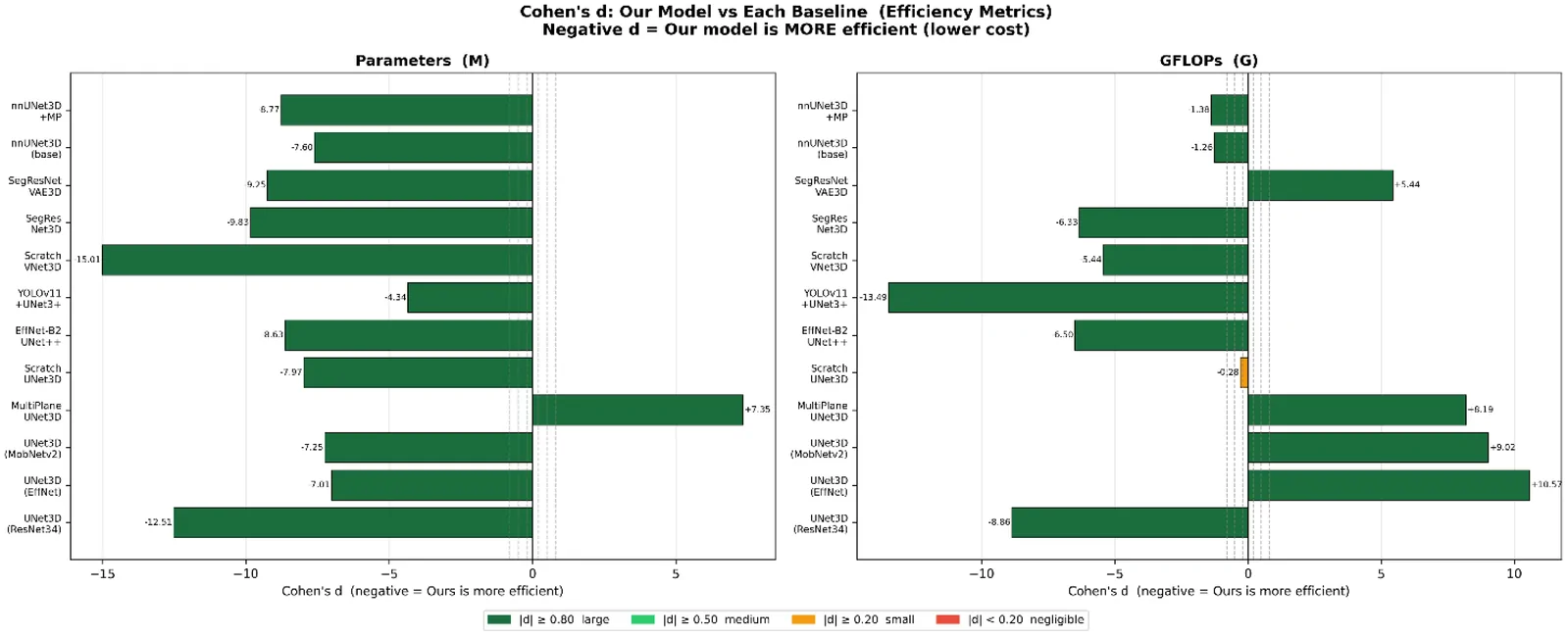
Cohen’s d test result of our model versus SOTA models across GFLOPs and parameters.

Our model exhibits superior parameter efficiency in the comparison graph shown in **Figure 13**, while sustaining competitive GFLOPs, with statistically significant effect sizes, rendering it the preferred selection for deployment under memory and storage limitations.

### 5.2 Qualitative analysis

**Figure 14:**
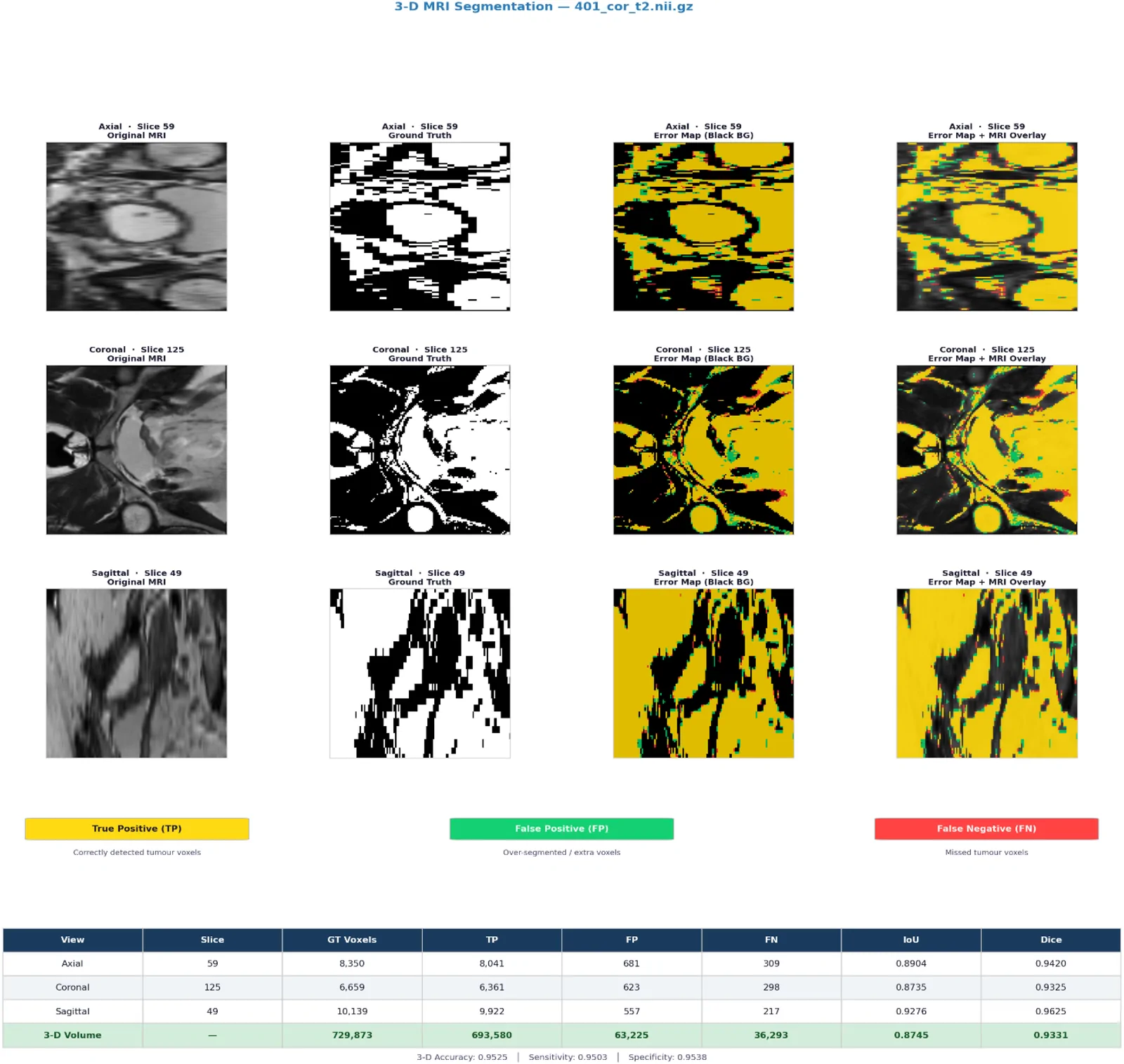
Qualitative visualization and error attribution analysis of a test case showing the original T2-weighted MRI, ground-truth mask, voxel-wise error map and error-overlay image in topmost row - axial view, middle row - coronal view and last row - sagittal view.

Despite the suggested framework demonstrating robust average segmentation performance, qualitative analysis of few test cases uncovered some problematic instances in which segmentation quality has slightly declined. **Figure 14** displays a test case showing IoU and dice scores, accompanied by axial, coronal and sagittal views, ground-truth masks and error maps delineating true positives, false positives and false negatives.

This qualitative analysis indicates that the primary segmentation deficiencies are located at the tumor boundary rather than the tumor core. The predominant sources of error in challenging cases are – (i) indistinct tumor margins, (ii) insufficient gray-scale contrast between the tumor and surrounding pelvic soft tissues, (iii) ambiguous tumor extent due to uncertainty or infiltration and (iv) anisotropic or thick slice MRI acquisition resulting in partial-volume effects and cross-plane inconsistency. Below test sample has a commendable overall overlap but revealed isolated boundary discrepancies in peripheral areas.

## 6 Conclusion and future work

In our study, we designed and assessed a multiplane-guided nn-UNet3D framework for automated MRI-based cervical cancer segmentation using the TCGA-CESC dataset. By combining axial, sagittal and coronal information within a volumetric learning paradigm, the model successfully captures long-range contextual dependencies while also improving robustness to anisotropic tumor presentation. The combination of multiplanar guidance and improved network components resulted in excellent segmentation accuracy of 95.04% and a high region-overlap performance, as evidenced by a Dice similarity coefficient of 93.52% and an Intersection over Union of 87.87%. These findings show that the proposed method produces precise and stable tumor delineation exceeding traditional single-plane, purely 2D architectures and baseline 3D networks by lowering segmentation ambiguity. Reliable and reproducible segmentation is critical for radiation planning and disease monitoring and automated multiplane-aware 3D segmentation has the potential to reduce inter-observer variability, reduce annotation burden and aid in more consistent treatment decisions.

Future work will focus on validating performance across various centers and MRI scanners to reduce domain-shift effects and increase deployment readiness. Exploring T1, T2, diffusion-based and contrast-enhanced sequences in future can leverage on complementary tissue contrast. Subsequent research may concentrate on boundary-aware supervision uncertainty-guided refinement and multimodal MRI integration to enhance robustness in challenging scenarios. Furthermore, investigating attention-based multiplane fusion or hybrid CNN-transformer to improve global context modeling can be focused in future work.

## Data Availability

Publicly available datasets were analyzed in this study. This data can be found here: https://www.cancerimagingarchive.net.
